# Rhizoplane microbiome: niche-specific recruitment and plant defense priming against bacterial wilt disease

**DOI:** 10.1093/plphys/kiag483

**Published:** 2026-07-08

**Authors:** Jiemeng Tao, Shizhou Yu, Peng Lu, Mengli Gu, Mengmeng Kong, Junjia Guo, Zhe Zhao, Huan Su, He Li, Jianfeng Zhang, Jingjing Jin, Peijian Cao

**Affiliations:** China Tobacco Gene Research Center, Zhengzhou Tobacco Research Institute of CNTC, Zhengzhou 450001, China; Beijing Life Science Academy, Beijing 102200, China; Molecular Genetics Key Laboratory of China Tobacco, Guizhou Academy of Tobacco, Guiyang 550081, China; China Tobacco Gene Research Center, Zhengzhou Tobacco Research Institute of CNTC, Zhengzhou 450001, China; Beijing Life Science Academy, Beijing 102200, China; China Tobacco Gene Research Center, Zhengzhou Tobacco Research Institute of CNTC, Zhengzhou 450001, China; School of Agricultural Sciences, Zhengzhou University, Zhengzhou 450001, China; China Tobacco Gene Research Center, Zhengzhou Tobacco Research Institute of CNTC, Zhengzhou 450001, China; China Tobacco Gene Research Center, Zhengzhou Tobacco Research Institute of CNTC, Zhengzhou 450001, China; Technology Center, China Tobacco Shandong Industrial Co., Ltd., Jinan 250014, China; China Tobacco Gene Research Center, Zhengzhou Tobacco Research Institute of CNTC, Zhengzhou 450001, China; China Tobacco Gene Research Center, Zhengzhou Tobacco Research Institute of CNTC, Zhengzhou 450001, China; China Tobacco Gene Research Center, Zhengzhou Tobacco Research Institute of CNTC, Zhengzhou 450001, China; Beijing Life Science Academy, Beijing 102200, China; China Tobacco Gene Research Center, Zhengzhou Tobacco Research Institute of CNTC, Zhengzhou 450001, China; Beijing Life Science Academy, Beijing 102200, China; China Tobacco Gene Research Center, Zhengzhou Tobacco Research Institute of CNTC, Zhengzhou 450001, China; Beijing Life Science Academy, Beijing 102200, China; School of Agricultural Sciences, Zhengzhou University, Zhengzhou 450001, China

## Abstract

The plant microbiome plays a pivotal role in host adaptation and disease suppression, yet niche-specific microbial responses to biotic stress, particularly within distinct plant compartments, remain poorly understood. Here, we revealed that bacterial wilt disease (BWD) induced pronounced niche-specific microbiome alterations in tobacco, with the rhizoplane (RP) emerging as a critical hub for beneficial microbial recruitment and defense coordination. Utilizing 16S and ITS amplicon sequencing across 6 distinct plant niches, we observed significantly enhanced bacterial diversity and a striking enrichment of potentially beneficial microbes in the RP under BWD stress. Eight potent antagonistic bacterial strains were isolated from this key niche, with *Stenotrophomonas* sp. ASV61 and *Chryseobacterium* sp. ASV172 demonstrating robust in vitro biocontrol potential and confirming in vivo plant resistance and growth promotion. We further elucidated the superior biocontrol mechanisms of *Chryseobacterium* sp. ASV172, attributing its superior efficacy to enhanced colonization and flexirubin-mediated antagonism. Crucially, plant transcriptomic profiling unveiled that these beneficial microbes engaged in a signaling dialogue with host plants, dynamically modulating defense hormone pathways. While *Ralstonia* alone manipulated host defenses by sustaining salicylic acid responses, antagonistic strains re-directed the plant toward robust jasmonic acid signaling, thereby restoring a more effective defense posture. Collectively, our findings underscore the disproportionate importance of the RP over the rhizosphere in assembling a resilient microbiome against soil-borne diseases, paving the way for targeted RP microbiome engineering strategies for sustainable disease management.

## Introduction

The plant microbiome, a complex consortium of microorganisms, is increasingly recognized as a pivotal architect of plant health, resilience, and adaptation to environmental challenges (Liu et al. 2020; Trivedi et al. 2020). These microbial communities inhabit distinct plant compartments, including the bulk soil (BS), rhizosphere (RS), rhizoplane (RP), endosphere, and phyllosphere (Compant et al. 2025). Each compartment possesses unique physicochemical properties and selective pressures, driving differential microbiome assembly and functional specialization (Philippot et al. 2013; Hardoim et al. 2015). Crucially, these microbial communities engage in sophisticated dialogues with their hosts, profoundly influencing plant growth and defense. Over the past decade, research has transitioned from cataloging microbial diversity to unraveling the intricate molecular mechanisms governing plant-microbe communication, particularly in the context of microbiome assembly and pathogen suppression (Su et al. 2024). Understanding these mechanisms is essential for harnessing the full potential of the plant microbiome to improve crop yields, enhance plant resilience, and promote sustainable agricultural practices.

Plant diseases represent a formidable biotic stress, exerting significant pressure on plant microbiomes and often triggering dynamic shifts that can lead to the recruitment of protective microbes (Gao et al. 2021; Bai et al. 2022; Wang and Song 2022). Plants can strategically modulate their microbial associations, employing a “cry for help” strategy by releasing specific signals to attract beneficial microorganisms while simultaneously inhibiting pathogens (Santoyo 2022; Liu et al. 2023). For example, tryptophan secreted by cucumber roots selectively stimulated the colonization of beneficial bacterium *Bacillus amyloliquefaciens* SQR9, which in turn suppressed soil-borne pathogens and steered the RS community toward a plant-beneficial composition (Liu et al. 2017). While connections between aboveground and belowground microbiomes are being established (Bai et al. 2015), a critical knowledge gap persists regarding how distinct plant compartments communicate and coordinate their responses to pathogen challenge. Specifically, the dynamic responses and inter-compartmental communication during disease progression, and the specialized roles of distinct plant-associated niches like the RP, remain poorly understood. The RP, representing the direct interface between root surface and the surrounding microbial community, is a crucial yet often under-investigated zone for mediating plant-microbe interactions under stress conditions (Zhang et al. 2017).

The deliberate inoculation of plants with beneficial or antagonistic microorganisms offers a potent avenue for sustainable disease management. These microbes can suppress pathogens through resource competition, antimicrobial production (Han et al. 2021; Yu et al. 2023), or by actively inducing systemic resistance (ISR) in host plants (Tao et al. 2024). Plant defense priming is a crucial phenomenon in plant immunity. It refers to a sensitized physiological state where prior perception of external biotic or abiotic signals primes the plant to exhibit a faster and stronger defense response upon subsequent pathogen challenge (Martinez-Medina et al. 2016). This enhanced readiness allows for more efficient and timely defense deployment without a constitutive increase in defense investment. In terms of microbial intervention, the, application of strains such as *Bacillus* spp. has shown efficacy against major pathogens like *Ralstonia solanacearum* and promoted plant growth by enhancing nutrient uptake and activating immune pathways (Wu et al. 2015; Sakthivel et al. 2019; Han et al. 2021). Emerging evidence also suggested that inoculations could reshape the resident plant microbiome, fostering a more conducive environment for plant health (Santoyo 2022). However, there is a scarcity of experimental evidence detailing how beneficial microbes orchestrate this microbiome restructuring and recruit novel beneficial partners to bolster plant immunity, particularly in specialized niches like the RP, which may serve as a primary site for recruiting protective agents.

Bacterial wilt disease (BWD), caused by the devastating soil-borne pathogen *R. solanacearum*, poses a critical threat to *Solanaceae* crops worldwide (Jiang et al. 2017). Infecting through root wounds and colonizing xylem vessels, *R. solanacearum* disrupts water transport, leading to wilting and mortality (Wei et al. 2018). Understanding the plant microbiome's role as a first-line defense mechanism against this root-initiated pathogen is crucial for developing effective control strategies (Durán et al. 2018; Wei et al. 2019). Given the importance of direct root-surface interactions in disease establishment and defense, our study focused on dissecting niche-specific microbiome dynamics, with a particular emphasis on the RP. We established a comprehensive, compartmentalized sampling framework across the soil-root-stem continuum in field-grown *Nicotiana tabacum*. By stratifying healthy and diseased plants into 6 discrete compartments—BS, RS soil, RP, root endosphere (RE), stem epidermis (SE), and stem xylem (SX)—and employing 16S and ITS amplicon sequencing, we uncovered pronounced niche-specific shifts and inter-compartmental communication patterns, highlighting the RP as a crucial hub for beneficial microbial recruitment and defense priming against *Ralstonia* infection. Our subsequent isolation and mechanistic investigation of key beneficial microbes enriched in the RP aimed to elucidate novel plant-microbe dialogues that primed host defense, paving the way for targeted microbiome engineering against BWD.

## Materials and methods

### Sample collection and processing

Field samples were collected in August 2021 from tobacco-growing regions of Fuquan (26°44′ 51' N, 107°30′26' E) and Zunyi (27°48′11' N, 107°42′47' E) in Guizhou province, China. The tobacco cultivar Yunyan 87, commonly planted by local farmers, was sampled at both locations. At each site, 6 biological replicates were collected for both healthy and diseased plants at the mature growth stage. Healthy plants were defined as those exhibiting no visible wilt symptoms. Diseased plants were characterized by severe wilt and brown discoloration of the vascular bundles, corresponding to an infection grade of 5 to 9. Two randomly selected individual plants were pooled to create each biological replicate. For each plant, samples of BS, RS soil, root tissue, and stem tissue were collected according to our previously described method (Tao et al. 2022, 2024). BS was collected at a distance of 20 cm radially from the root at a depth of 15 cm. RS soil was obtained by brushing the remaining soil from the root after shaking off loosely attached soil and rocks. Stem sample was collected 20 cm above the root-soil interface. All collected samples (root, stem, RS soil, and BS) were immediately transported to the laboratory on dry ice and stored at −80 °C until further processing.

**Figure 1:**
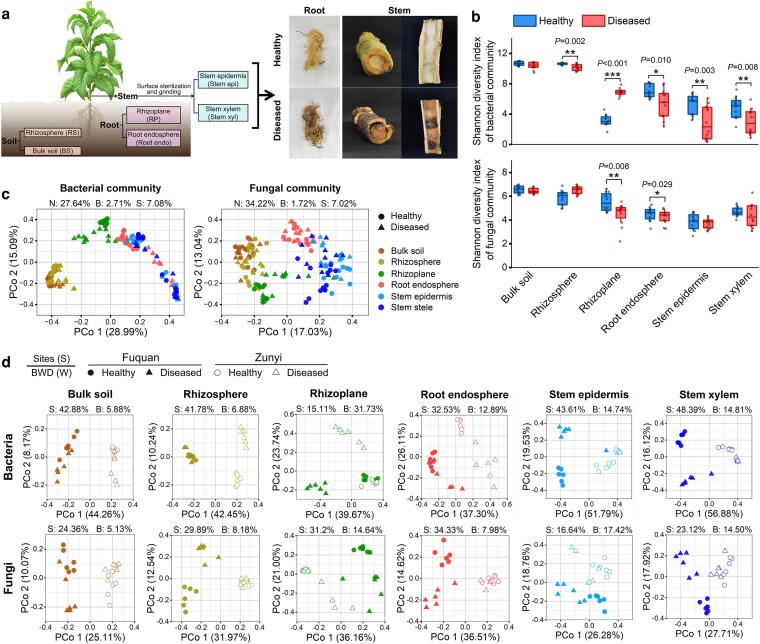
Assembly of bacterial and fungal communities of healthy and diseased plants. a) The 6 compartment niches of a tobacco plant and the root and stem phenotypes of healthy and diseased plants. b) Shannon diversity index of bacterial and fungal communities in the 6 compartments of healthy and diseased plants. c) Principal coordinate analysis (PCoA) based on Bray–Cutis dissimilarity matrices and PERMANOVA, showing the importance of the compartment niche (N), BWD (B) and sampling site (S) on the bacterial (left) and fungal (right) community structures. d) PCoA based on Bray–Cutis dissimilarity matrices and PERMANOVA of bacterial and fungal communities in each compartment niche.

In the laboratory, root and stem samples were processed to form different microbial fractions. Root samples were placed in sterile 50 mL tubes containing 15 mL of sterile phosphate buffered saline (PBS) solution and subjected to ultrasonic agitation at 40 kHz for 1 min. The resulting washing solution was collected and centrifuged at 12,000 rpm for 15 min to obtain RP samples. The remaining roots and stems were surface sterilized 3 times by sequential washes in 75% ethanol (5 min), 1% sodium hypochlorite (5 min), 75% ethanol (30 s), and sterile water. Surface-sterilized stems were then separated into SE and SX compartments. Sterilized root, SE, and SX samples were cut into small pieces and stored at −80 °C prior to DNA extraction. In total, each plant sample was divided into 6 compartments: BS, RS, RP, RE, SE, and SX (Fig. 1a). Distinct phenotypic differences in roots and stems were observed between healthy and diseased samples (Fig. 1a).

### DNA extraction, amplicon sequencing and data processing

Total genomic DNA was extracted using the Mag-Bind Soil DNA Kit (Omega Biotek Inc., Doraville, GA, USA) according to the manufacturer's instructions. For BS, RS soil, and RP microbial enrichments, ∼0.5 g of sample was directly subjected to extraction. For RE, SE, and SX samples, ∼5 g of tissue was first cryogenically ground using a sterile mortar and pestle with liquid nitrogen. Subsequently, ∼0.5 g of the resulting tissue powder was then used for DNA extraction following the same kit protocol.

The V5-V7 hypervariable region of bacterial 16S rRNA gene was amplified using primers 799F (5′-AACMGGATTAGATACCCKG-3′) and 1193R (5′-ACGTCATCCCCACCTTCC-3′), and fungal ITS1 region was amplified using primers ITS1F (5′-CTTGGTCATTTAGAGGAAGTAA-3′) and ITS2 (5′-GCTGCGTTCTTCATCGATGC-3′). PCR reactions were performed in a 25 *μ*L volume containing 12.5 *μ*L Taq Master Mix (Vazyme, China), 0.5 *μ*L of each primer (10 *μ*m), 2.0 *μ*L template DNA (∼25 ng/*μ*L), and nuclease-free water to adjust the volume. The amplification conditions for bacteria were: initial denaturation at 94 °C for 2 min; 30 cycles of 94 °C for 30 s, 55 °C for 30 s, and 72 °C for 45 s; and a final extension at 72 °C for 5 min. For fungi, the conditions were: 94 °C for 5 min; 35 cycles of 94 °C for 30 s, 56.5 °C for 30 s, and 72 °C for 30 s; and a final extension at 72 °C for 7 min. High-throughput sequencing was carried out on the Illumina MiSeq platform following a paired-end protocol (2 × 250 bp). Raw sequencing reads were demultiplexed based on sample-specific barcodes and primer sequences, and subsequently processed using QIIME 2 (Bolyen et al. 2019). Specifically, the DADA2 plugin was employed for quality control, denoising, and chimera filtering, generating a table of amplicon sequence variants (ASVs). Taxonomic assignment of bacterial and fungal ASVs was performed using the SILVA (v138) (Yilmaz et al. 2014) and UNITE (v2021.5.10) (Nilsson et al. 2019) databases, respectively.

To distinguish endophytic bacterial amplicons from plant organelle-derived amplicons, the primer pair 799F/1193R was chosen because it is known to have mismatches with plant mitochondrial 16S rRNA and greatly minimize amplification of chloroplast 16S rRNA, thereby reducing the amplification of plant-derived sequences during PCR. In addition, after taxonomic assignment, all bacterial ASVs classified as Chloroplast or Mitochondria were removed from the dataset. For fungi, ASVs identified as Plantae or Protista were excluded from downstream analyses. This 2-pronged approach, combining primer design to minimize plant DNA co-amplification and subsequent bioinformatic filtering, ensured a robust and reliable identification of endophytic microbial communities.

### Isolation and culture of bacterial strains from diseased RP samples

Bacterial strains were isolated and cultivated from diseased RP samples following a modified high-throughput cultivation method based on Zhang et al. (2021a). Briefly, 0.2 g of RP microbial enrichment was transferred into a 100 mL sterile shake flask containing 50 mL of 1×PBS. The mixture was vortexed for 10 min and incubated at room temperature for 30 min to release the bacteria from the RP samples. The resulting bacterial suspension was serially diluted, dispensed into 96-well microtiter plates, and cultured in 1/10 tryptic soy broth (TSB) at 28 °C for 2 wk. To identify cultured isolates, the 16S rDNA V5 to V7 region (corresponding to the region amplified in the 16S metabarcoding analysis) was amplified using primers 799F and 1193R. Isolates were indexed using a 2-step PCR approach with primers containing well- and plate-specific barcodes to amplify the V5 to V7 region. Cultured bacterial sequences were compared with the ASVs obtained from the corresponding RP 16S metabarcoding data. Isolates exhibiting >99% gene identity to an ASV were considered representative of that ASV. Each ASV identified from cultured bacteria was selectively cultivated and purified on the 1/2 trypticase soy agar (TSA) medium. The taxonomic identity of purified isolates was confirmed by Sanger sequencing of the nearly full-length 16S rRNA gene using primers 27F and 1492R. Multiple sequence alignment was performed using MEGA X software (Kumar et al. 2018), and a phylogenetic tree was constructed using the Neighbor-Joining method.

### In vitro antagonistic activities of bacteria against *R. solanacearum*

The antagonistic activity of isolated bacterial strains against *R. solanacearum* were evaluated using a plate confrontation assay (Levin et al. 2019). Briefly, 100 *μ*L of pathogen suspension (OD_600_ = 0.5) was evenly spread onto TSA plates. After surface drying, an 8 mm diameter hole was aseptically created at the center of each plate using a sterile hole punch. Subsequently, 100 *μ*L of each bacterial isolate's fermentation broth (OD_600_ = 0.5) was added into the hole. Plates were incubated at 30 °C for 48 h, and the presence and diameter of any resulting inhibition zone around the hole were recorded as a measure of antagonism. The experiment was performed in triplicate.

### Glasshouse experiments on plant growth and disease suppression by antagonistic bacteria

Two bacterial strains, *Stenotrophomonas* sp. ASV61 and *Chryseobacterium* sp. ASV172, were selected for glasshouse-based biocontrol evaluation of BWD based on their strong antagonistic activity against *R. solanacearum* in vitro. A completely randomized design with 6 treatments was implemented: (i) Control (CK), plants treated with sterile distilled water; (ii) ASV61, plants inoculated with *Stenotrophomonas* sp. ASV61 only; (iii) ASV172, plants inoculated with *Chryseobacterium* sp. ASV172 only; (iv) Rs, plants inoculated with *R. solanacearum* only; (v) ASV61 + Rs, plants co-inoculated with *Stenotrophomonas* sp. ASV61 and *R. solanacearum*; (vi) ASV172 + Rs, plants co-inoculated with *Chryseobacterium* sp. ASV172 and *R. solanacearum*. The bacterial suspensions were prepared according to the following protocol: First, each bacterial isolate was streaked onto a TSA plate and incubated for 3 d. Subsequently, a single colony was selected from each plate and cultured in TSB liquid medium at 28 °C with shaking at 180 rpm for 3 d. Finally, the bacterial suspensions were diluted to an optical density of OD_600_ = 0.5 prior to experimental use. *N. tabacum* (cv. Hongda) seeds were surface sterilized by immersion in 10% sodium hypochlorite for 10 min, followed by rinsing with sterile distilled water. Sterilized seeds were germinated on 1/2 Murashige and Skoog (MS) agar plates under controlled conditions. Twenty-day-old seedlings were transplanted individually into pots (0.5 L, diameter = 10 cm) filled with a substrate composed of peat and vermiculite at a volume ratio of 7:3. For treatments involving ASV61 and ASV172, 10 mL suspension of each bacterial strain (OD_600_ = 0.5) was applied to the soil around the roots 10 d after transplanting. Seven days later, treatments containing Rs received 5 mL of *R. solanacearum* suspension (OD_600_ = 0.5). The control group received the same volume of distilled water. Each treatment consisted of 15 replicated plants. Plants were maintained in a growth chamber under controlled environmental conditions (16 h light/8 h dark photoperiod, 25 °C). Disease incidence was recorded at 10 d after pathogen inoculation, and plant growth parameters were measured. The disease index was rated using a 5-grade scale: Grade 0: No symptoms, Grade 1: 1% to 25% of leaves wilted, Grade 2: 26% to 50% of leaves wilted, Grade 3: 51% to 75% of leaves wilted, Grade 4: 76% to 100% of leaves wilted (Roberts et al. 1988). Three RS samples were randomly collected for amplicon sequencing, with corresponding root samples for RNA sequencing (transcriptome analysis) and phytohormones detection.

### RNA-seq of tobacco roots after inoculation with antagonistic bacteria

Total RNA was extracted from fresh root tissues using TRIzol Reagent (Invitrogen). The integrity and purity of the RNA were assessed using an Agilent 2100 Bioanalyzer (Agilent Technologies, CA, USA) and a NanoDrop 2000 spectrophotometer (Thermo Fisher Scientific, USA), respectively. After sequencing, reads containing adapters, poly-N, and low quality were removed to obtain clean reads (Chen et al. 2018). Clean reads were aligned to the tobacco reference genome (Wang et al. 2024) using HISAT2 (Kim et al. 2015). The calculation of fragments per kilobase of transcript per million mapped reads (FPKM) for each gene was performed, and the read counts of each gene were acquired using HTSeq-count (Roberts et al. 2011; Simon et al. 2015). Differential expression analysis of genes was performed using the DESeq2 method with a *P* < 0.05 and fold change >2 as the threshold (Roberts et al. 2011). Gene ontology (GO) (The Gene Ontology Consortium 2019) pathway enrichment analysis of the differentially expressed genes (DEGs) were determined using the R package clusterProfiler v4.14.4.

### Quantification of defense-related genes

A total of 1 *μ*g of high-quality RNA was reverse transcribed into cDNA using the PrimeScript RT Reagent Kit (Takara Bio, Japan). Subsequently, quantitative reverse transcription PCR (qRT-PCR) was performed in 15 *μ*L reaction volumes containing 1 *μ*L specific primers (10 *μ*m), 7.5 *μ*L SYBR Green qPCR mix, 2 *μ*L template cDNA (50 ng/*μ*L), and 4.5 *μ*L dH_2_O. Gene-specific primers were designed using Primer 5.0 software and commercially synthesized by BGI Genomics Co., Ltd, China. Amplification specificity was validated through single-peak dissociation curves analysis from melting curve analysis. Eight defense-related genes associated with salicylic acid (SA) (*NtICS2*, *NtNPR1*, *NtPR1*, and *NtTGA2*) and jasmonic acid (JA) (*NtAOC3*, *NtAOS*, *NtJAO3*, and *NtLOX3*) signaling pathways were analyzed. The relative expression levels of these genes were quantified via the 2^−ΔΔCt^ method, with *GAPDH* serving as the internal reference. All primer sequences were provided in Table S1. Three technical replicates were analyzed per biological replicate.

### Phytohormones detection and analysis

Ultra performance liquid chromatography (ExionLC AD) combined with Tandem Mass Spectrometry (MS/MS) (QTRAP 6500+) was employed to detect the contents of phytohormones in tobacco roots. Compound identification was achieved through spectral library matching against authenticated reference standards. Quantitative measurements employed multiple reaction monitoring mode with signal acquisition controlled at 2.5 ms dwell time per transition. Raw MS/MS data were processed through MultiQuant 3.0.3 software for peak integration and analyte quantification. Chromatographic peaks corresponding to the tested substances in different samples were integrated and corrected. Total ion chromatograms were used to assess the overall quality of the chromatographic separation and the stability of the analytical system. The coefficients of variation were calculated to evaluate the reproducibility of the measurements.

### Whole genome sequencing, assembly, and annotation of *Chryseobacterium* sp. ASV172

The complete genome of *Chryseobacterium* sp. ASV172 was sequenced on the Illumina MiSeq platform (2 × 150 bp paired-end reads) by Novogene Technology Co. Ltd (Tianjin, China). Raw sequence quality was checked using Fastp (v0.23.1) (Chen 2023). De novo assembly of the high-quality scaffolds was carried out using SPAdes (v3.9.0) (Bankevich et al. 2012). The identification of coding genes and functional annotation were performed by querying a series of databases, including the non-redundant database, SwissProt (Bairoch and Apweiler 2000), Clusters of Orthologous Groups (COG) (Koonin 2002), Kyoto Encyclopedia of Genes and Genomes (KEGG) (Minoru et al. 2008), and GO. The antiSMASH 7.0 (Blin et al. 2023) database was used to analyze biosynthetic gene clusters (BGCs) responsible for secondary metabolite biosynthesis.

### Statistical analyses

All statistical analyses were conducted using packages in R software (v4.4.3). The “vegan” package (v2.7-1) was used to calculate the alpha diversity indices, including the Shannon index and Chao1 richness. Multiple comparisons between the alpha index across different compartments or healthy conditions were assessed with the Kruskal–Wallis rank-sum test. Principal coordinate analysis (PCoA) based on the Bray–Curtis dissimilarity was used to characterize the beta diversity. The contribution of different factors on community dissimilarity was examined with PERMANOVA (permutational multivariate analysis of variance) using the Adonis function in the “vegan” package. Differential analysis of ASVs' relative abundance between healthy and diseased samples was performed using the DESeq2 method. One-way analysis of variance (ANOVA) followed by the LSD test was used to determine the statistical significance between inoculation-treatments.

## Results

### BWD exerted niche-specific effects on plant microbiome assembly

A total of 8,961,704 bacterial and 9,269,711 fungal high-quality reads were extracted from 144 samples. The reads were clustered into 7,745 bacterial and 2,205 fungal ASVs, respectively. Subsequent rarefaction analysis was performed at standardized sequencing depths of 26,515 reads for bacterial communities and 30,744 reads for fungal communities to ensure comparable alpha diversity assessments. Alpha diversity revealed a general trend of decreasing bacterial and fungal diversity from soil to plant tissues, as indicated by both the Shannon diversity index (Fig. S1a) and Chao1 richness (Fig. S1b). Notably, bacterial communities demonstrated greater sensitivity to BWD than fungal communities, showing more pronounced variations in alpha diversity metrics (Figs. 1b and S1c). Specifically, bacterial alpha diversity was significantly reduced (*P* < 0.05) in diseased RS, RE, SE, and SX compartments compared with healthy plants. Conversely, the RP of diseased plants exhibited significantly higher bacterial diversity than healthy plants (*P* < 0.05, Figs. 1b and S1c). For fungal alpha diversity, significant reductions of Shannon diversity were observed only in diseased RP and RE, while Chao1 richness was significantly lower only in diseased SE compared with healthy plants (*P* < 0.05, Figs. 1b and S1c). Furthermore, BWD-induced alterations in bacterial alpha diversity displayed stronger spatial consistency across experimental sites compared with fungal communities (Fig. S1d and e).

To assess the relative contribution of plant niche, BWD, and sampling site on microbial community assembly, PCoA and PERMANOVA analyses were performed. The results showed that the niche explained the largest variations for both bacterial (27.64%, *P* = 0.001) and fungal (34.22%, *P* = 0.001) communities, followed by the sampling site (bacteria 7.08%, fungi 7.02%, *P* = 0.001 for both) and BWD (bacteria 2.71%, *P* = 0.002; fungi 1.72%, *P* = 0.009) (Fig. 1c and Table S2). BWD exhibited compartment-specific effects, demonstrating stronger impacts on microbial communities in plant compartments niches (RP, RE, SE, and SX) than those in soil niches (BS and RS) (Fig. 1d). Bacterial communities showed greater sensitivity to BWD than fungal communities across most compartments, with particularly pronounced effects in the RP (bacteria 31.73%, fungi 14.64%, *P* = 0.001 for both). This pattern was consistent in BS (bacteria 5.88%, fungi 5.13%), RE (bacteria 12.89%, fungi 7.98%), and SX (bacteria 14.81%, fungi 14.50%) (Fig. 1d and Tables S3 and S4).

**Figure 2:**
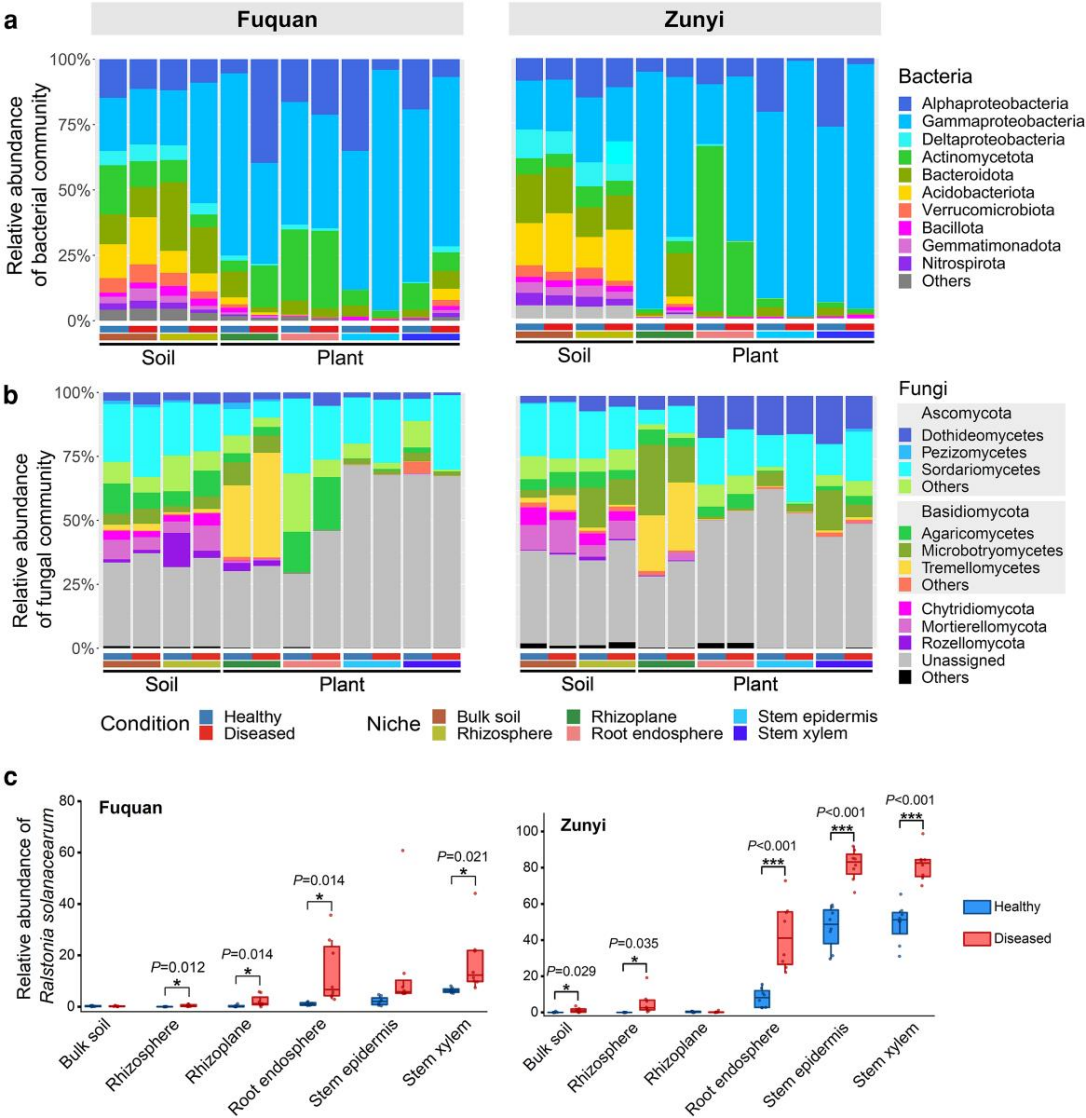
Taxonomic composition of bacterial and fungal communities of healthy and diseased plants. The relative abundance of main bacterial (a) and fungal (b) phyla/class in 6 compartment niches of healthy and diseased plants at 2 study sites (Fuquan and Zunyi). The top 10 most abundant bacterial or fungal phyla/class are shown and other less abundant taxa are grouped into “Others.” c) The relative abundance of *R. solanacearum* in 6 compartment niches of healthy and diseased plants at 2 study sites (Fuquan and Zunyi).

Taxonomic classification revealed distinct differences in the dominant phyla between plant and soil niches. In bacterial communities, *Pseudomonadota* including *Alphaproteobacteria* and *Gammaproteobacteria* dominated plant compartments, while soil niches exhibited higher abundances of *Bacteroidota*, *Acidobacteriota*, *Verrucomicrobiota*, and *Gemmatimonadota* (Fig. 2a). In fungal communities, the relative abundance of *Tremellomycetes* belonging to *Basidiomycota* was significantly higher in the RP, while *Chytridiomycota*, *Mortierellomycota*, and *Rozellomycota* were more abundant in BS and the RS (Fig. 2b). BWD altered both bacterial and fungal community composition in plant compartment niches, particularly in the RP (Fig. 2a and b). Specifically, the relative abundance of the bacterial *Alphaproteobacteria* and *Actinomycetota* (Fig. 2a) and the fungal *Tremellomycetes* (Fig. 2b) was significantly higher in diseased RP samples than those in healthy ones at both experimental sites. Furthermore, the relative abundance of the pathogen *R. solanacearum* was markedly elevated in diseased samples compared with healthy samples across most niches (Fig. 2c). With the exception of BS from Fuquan and the RP from Zunyi, all other niches at both sites exhibited an enrichment of *R. solanacearum* in diseased samples. Overall, the proportion of *R. solanacearum* was significantly higher within plant tissues compared with soil niches. Notably, in the diseased stems from Zunyi, *R. solanacearum* accounted for ∼80% of the bacterial community, indicating a substantial colonization in the stem tissue (Fig. 2c).

### Potential beneficial microbes were recruited in diseased plants

**Figure 3:**
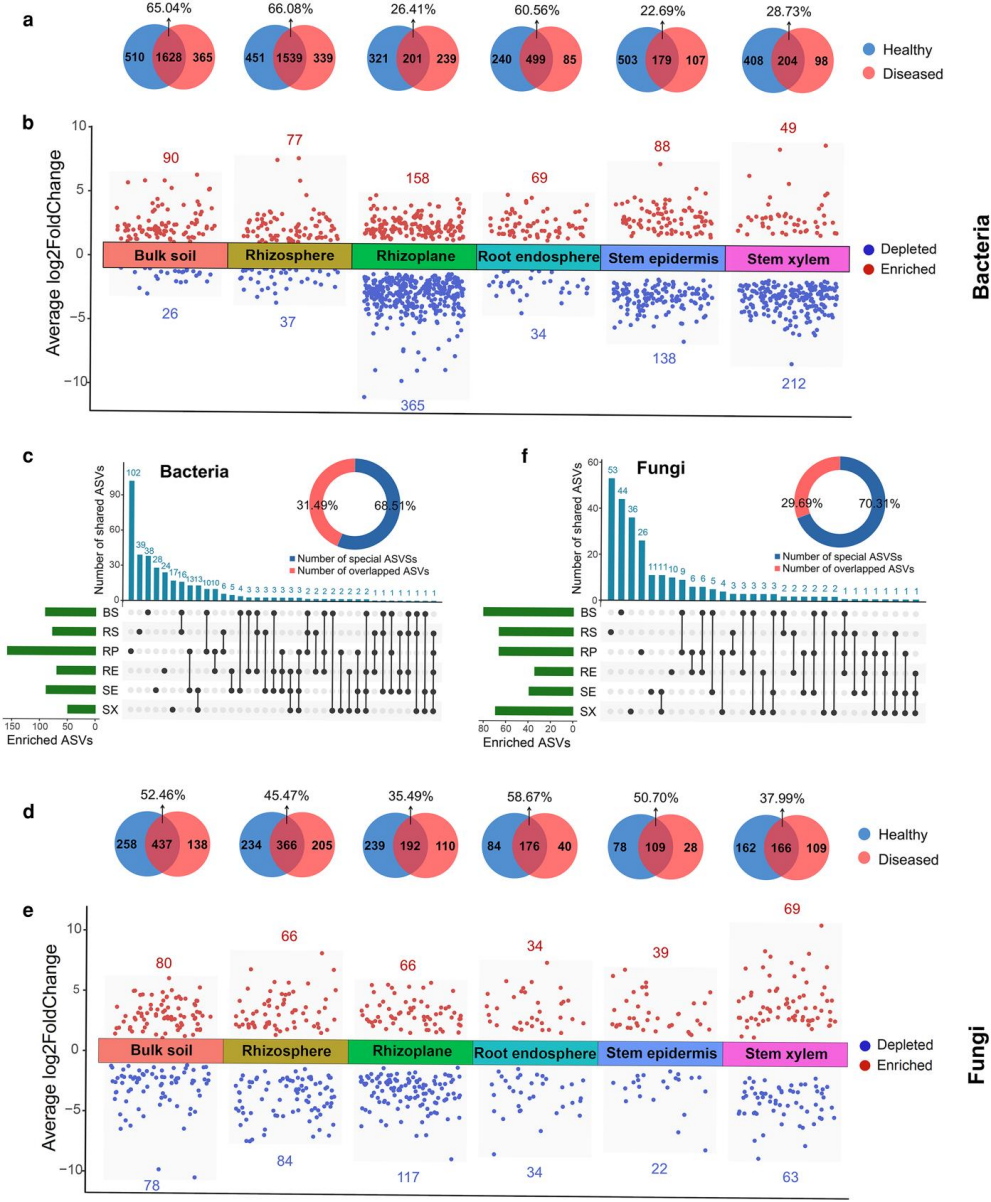
The enrichment and depletion patterns of bacterial and fungal communities in the diseased plants compared with the healthy ones. Venn diagrams showing the distribution of bacterial (a) and fungal (d) ASVs between healthy and diseased samples of different ecological niches. Volcano plots showing bacterial (b) and fungal (e) ASVs enriched or depleted in different compartment niches of diseased plants. Each circle represents a unique ASV (ASVs abundance >0.001%, *P* < 0.05). Upset diagram illustrating the overlap of enriched bacterial (c) and fungal (f) ASVs across 6 compartment niches of diseased plants. The pie chart showing the proportion of enriched ASVs specific to or shared between different niches.

The analysis of ASVs composition revealed that healthy and diseased samples shared 22.69% to 66.08% of bacterial ASVs (Fig. 3a) and 35.49% to 58.67% of fungal ASVs (Fig. 3d) across the 6 niches. The RP exhibited a significantly lower proportion of shared ASVs between healthy and diseased samples (26.41% for bacteria, 35.49% for fungi). This distinct microbial partitioning in the RP corresponded to a greater number of ASVs being either enriched or depleted compared with the other niches (Fig. 3b and 3e, Tables S5 and S6), suggesting a more pronounced shift in microbial community structure in this niche in response to disease. Specifically, the diseased RP exhibited significant enrichment of 158 bacterial ASVs (Fig. 3b) and 66 fungal ASVs (Fig. 3e) (FDR adjusted *P* < 0.05, Wilcoxon rank sum test). The bacterial ASVs enriched in diseased RP were predominantly affiliated with the phyla of *Pseudomonadota* (63.29%) and *Actinomycetota* (25.32%), encompassing several potentially beneficial genera such as *Bosea*, *Chryseobacterium*, *Massilia*, *Microbacterium*, *Pseudomonas*, *Rhizobium*, *Sphingomonas*, and *Streptomyces* (Table S5). In contrast, a large proportion of the enriched fungal ASVs (over 60%) in the diseased RP could not be classified at the genus level, highlighting substantial taxonomic uncertainty within fungi (Table S6).

Analysis of niche-specific ASV enrichment revealed that a majority of both bacterial (68.51%) (Fig. 3c) and fungal (70.31%) ASVs (Fig. 3f) were uniquely enriched in a single niche. Within the bacterial community, the diseased RP harbored the highest number of uniquely enriched ASVs (102), significantly exceeding those observed in other niches (Fig. 3c). The distribution of enriched fungal ASVs demonstrated a greater spatial heterogeneity, with 44, 53, 26, 10, 11, and 36 uniquely enriched ASVs identified in BS, RS, RP, RE, SE, and SX, respectively (Fig. 3f). No ASVs were commonly enriched across all 6 niches, with overlap observed only between 2 or 3 niches.

**Figure 4:**
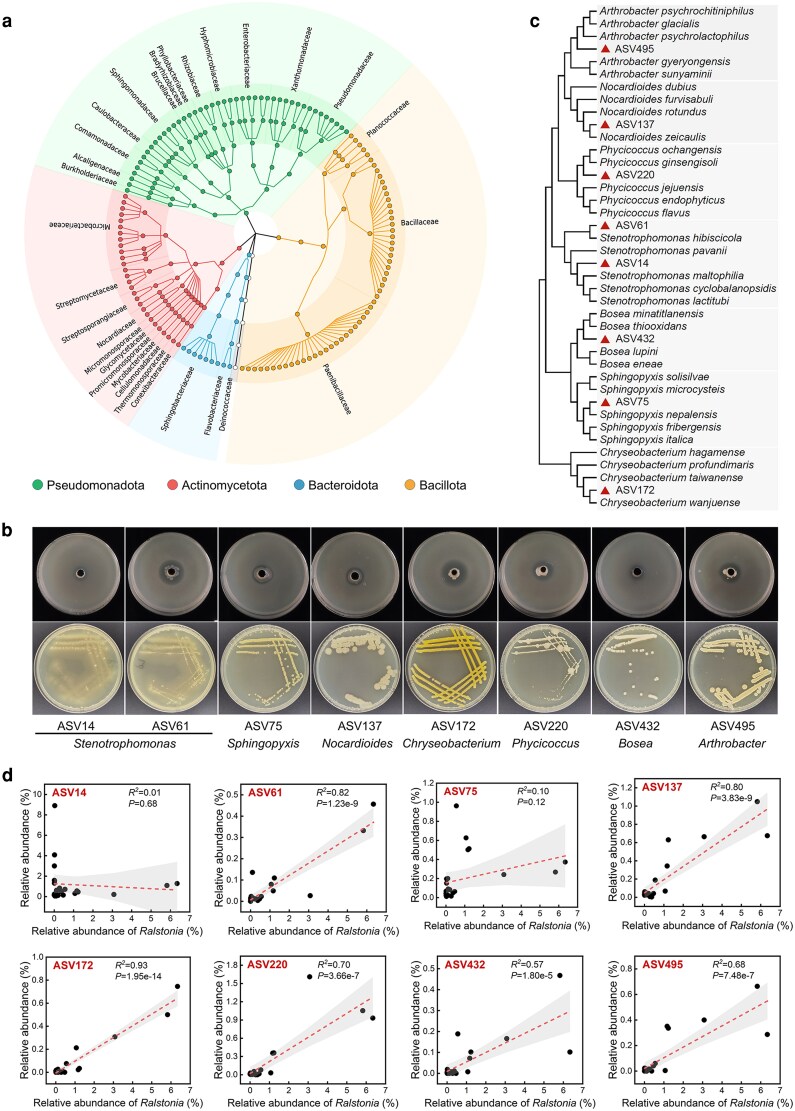
Isolation and identification of bacterial strains against the pathogen *R. solanacearum* from diseased RP samples. a) Cladogram showing the taxonomic distribution of isolated culturable bacteria in diseased RP samples. b) The antagonistic activities of 8 bacterial strains in plate confrontation experiments and their colonial morphology on plates. c) Phylogenetic analysis based on 16S gene sequence showing the phylogenetically close taxa of the 8 antagonistic strains. d) The correlation analysis between the pathogen *R. solanacearum* and the 8 antagonistic strains.

Given the pronounced structural shifts in the RP bacterial community under disease conditions, we isolated bacteria from the diseased RP samples using a high-throughput cultivation approach. A total of 147 cultivable bacterial isolates were obtained and identified by 16S rRNA gene sequencing (Fig. 4a). Comparative analysis of the V5 to V7 region sequences against amplicon sequencing data assigned these isolates to 118 distinct ASVs spanning 50 genera. Plate confrontation assays against *R. solanacearum* identified 8 antagonistic strains: ASV14, ASV61, ASV75, ASV137, ASV172, ASV220, ASV432, and ASV495 (Fig. 4b). Particularly, ASV61 and ASV172 exhibited the strongest antagonistic activities (Fig. 4b), with inhibition zones of 26.7 ± 0.70 and 28.6 ± 1.39 mm, respectively (Fig. S2). Phylogenetic analysis of 16S rRNA gene sequences identified these antagonistic strains as closely related to *Stenotrophomonas maltophilia*, *Stenotrophomonas hibiscicola*, *Sphingopyxis nepalensis*, *Nocardioides zeicaulis*, *Chryseobacterium wanjuense*, *Phycicoccus ginsengisoli*, *Bosea thiooxidans*, and *Arthrobacter psychrolactophilus* (Fig. 4c). Importantly, the 8 antagonistic strains were found to be significantly enriched in diseased RP (Fig. 3b and Table S5). Furthermore, most of them showed a positive correlation with the relative abundance of *Ralstonia* in the RP. This correlation was particularly strong and significant for *Stenotrophomonas* sp. ASV61 (*R*^2^ = 0.82, *P* = 1.23e−9) and *Chryseobacterium* sp. ASV172 (*R*^2^ = 0.93, *P* = 1.95e−14) (Fig. 4d). Collectively, these results suggested that plants might recruit specific beneficial microbes to suppress pathogens.

### Antagonistic strains showed significant biocontrol effects on plants

**Figure 5:**
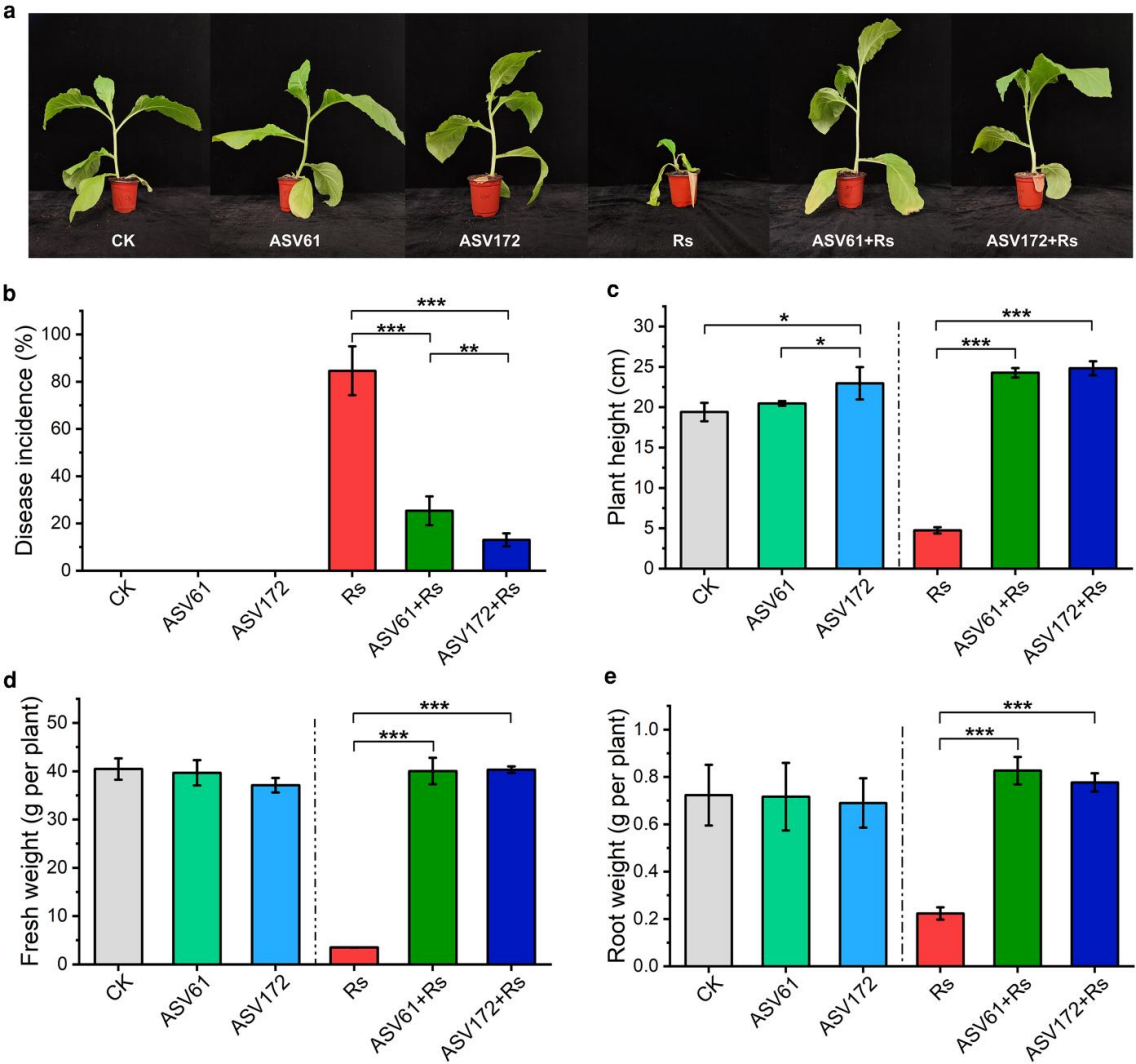
Effects of inoculation with antagonistic strains *Stenotrophomonas* sp. ASV61 and *Chryseobacterium* sp. ASV172 on tobacco growth and survival. a) Representative phenotypes of tobacco after inoculating antagonistic strains and *R. solanacearum* in glasshouse experiments. CK, no inoculation; ASV61, inoculation with ASV61 only; ASV172, inoculation with ASV172 only; Rs, inoculation with *R. solanacearum* only; ASV61 + Rs, inoculation with ASV61 and *R. solanacearum*; ASV172 + Rs, inoculation with ASV172 and *R. solanacearum*. b) Disease incidence was scored at 10 d after *R. solanacearum* inoculation. Plant height (c), aboveground fresh weight (d) and root weight (e) of tobacco 10 d after *R. solanacearum* inoculation. Asterisks indicate significant differences between treatments (*P < 0.05; **P < 0.01; ***P < 0.001). The statistical comparisons shown in (c to e) were based on 3 independent biological replicates per treatment.

To elucidate the potential mechanisms of biocontrol, *Stenotrophomonas* sp. ASV61 and *Chryseobacterium* sp. ASV172 were evaluated for their ability to control BWD in tobacco. In the absence of the *Ralstonia* pathogen, plants inoculated with ASV172 showed a statistically significant increase in height compared with those inoculated with ASV61 or the control (Fig. 5a and c). Plate experiments further confirmed the growth-promoting effects of both ASV61 and ASV172 on tobacco seedlings, as evidenced by increased fresh weight, plant height, root length, and leaf width (Fig. S3). Under pathogen challenge, both ASV61 and ASV172 significantly enhanced plant resistance to *R. solanacearum* and promoted plant growth (Fig. 5). Symptoms gradually developed over the 10-d observation period, starting at ∼2 to 3 d post inoculation (dpi). Specifically, at 10 dpi with *R. solanacearum*, plants co-inoculated with ASV61 or ASV172 showed a significant reduction in wilt disease incidence (25.38% and 13.08%, respectively) compared with plants inoculated with *R. solanacearum* alone (84.62%) (*P* < 0.001; Fig. 5b). Similarly, disease severity was markedly lower in the co-inoculated treatments (ASV61 + Rs: 14.10; ASV172 + Rs: 11.54) than in the pathogen-only treatment (55.13), indicating that both strains conferred significant protection against *R. solanacearum* infection (*P* < 0.001; Fig. S4). Concurrently, co-inoculated plants showed 5.13- and 5.25-fold increases in plant height (Fig. 5c), 11.42- and 11.50-fold enhancements in fresh weight (Fig. 5d), and 3.70- and 3.48-fold elevations in root weight (Fig. 5e) compared with pathogen-only plants (*P* < 0.001). These results demonstrated effective biocontrol and growth promotion by both strains under pathogen stress, with ASV172 exhibiting a stronger disease control effect than ASV61.

### Antagonistic strains promoted plant fitness by shaping RS microbiome and producing antimicrobial metabolites

**Figure 6:**
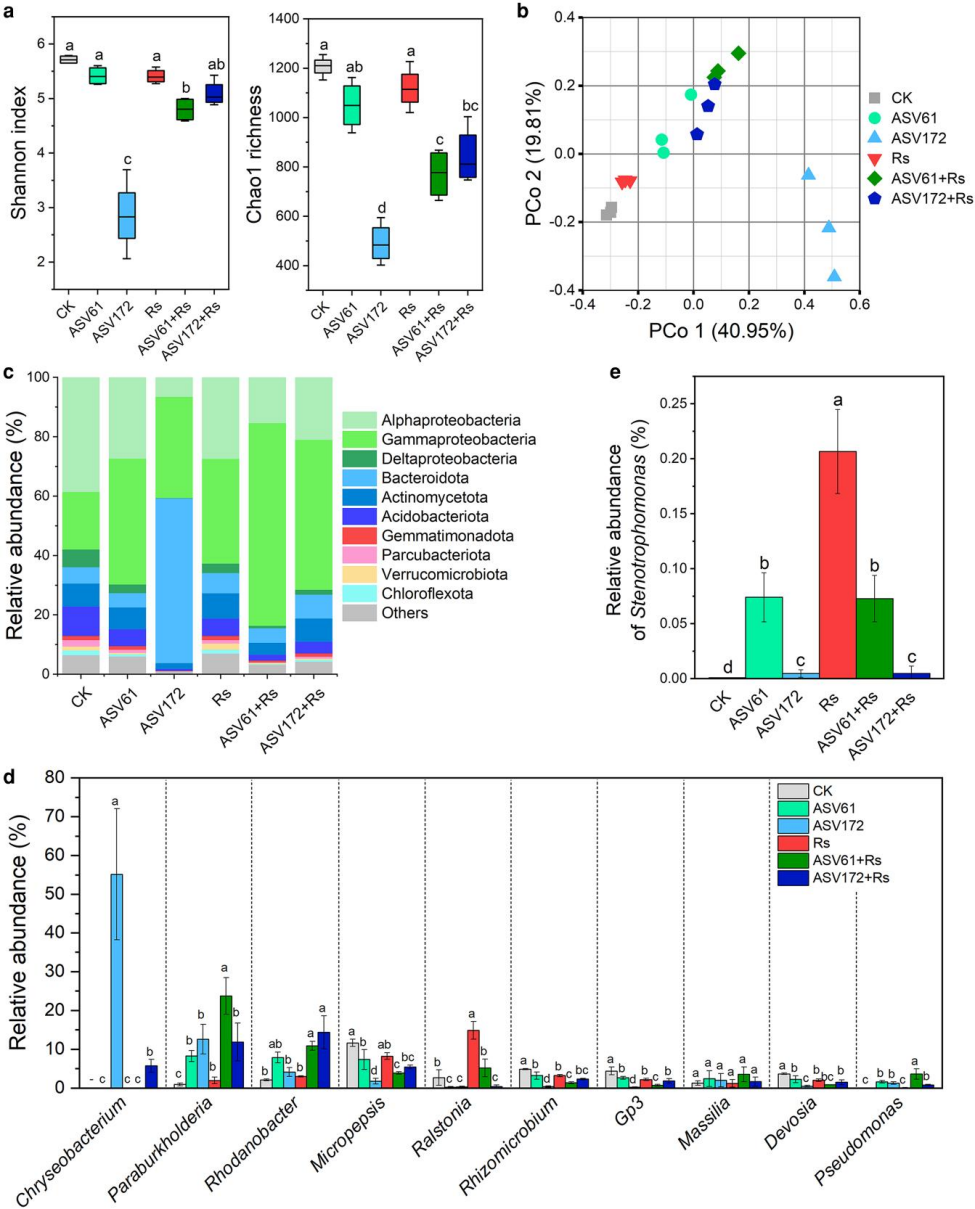
Effects of inoculation with antagonistic strains *Stenotrophomonas* sp. ASV61 and *Chryseobacterium* sp. ASV172 on tobacco RS bacterial community. a) Shannon diversity and Chao1 richness indices of RS bacterial communities under different treatments. b) PCoA based on Bray–Cutis dissimilarity matrices illustrating structural divergence of bacterial communities among treatments. c) Taxonomic composition of RS bacteria at the phylum/class level. The top 10 most abundant phyla/class are shown and less abundant taxa are grouped into “Others.” d) Relative abundance of the top 10 most abundant bacterial genera in each treatment. e) Relative abundance of *Stenotrophomonas* in each treatment. Different lowercase letters above bars indicated statistically significant differences (*P* < 0.05) as assessed by 1-way ANOVA followed by Tukey's multiple comparison test.

The effects of *Stenotrophomonas* sp. ASV61 and *Chryseobacterium* sp. ASV172 on the RS microbiome were investigated. Both ASV61 and ASV172 significantly altered the RS microbiome, leading to a general reduction in alpha diversity with or without pathogen inoculation. Specifically, Shannon diversity and Chao1 richness were significantly lower in the ASV172 treatment compared with the control and ASV61 treatment in the absence of the pathogen (*P* < 0.05) (Fig. 6a). PCoA analysis revealed significant shifts in bacterial community composition across treatments (Fig. 6b; PERMANOVA, *P* < 0.01). Notably, ASV172 treatment resulted in a distinct community structure compared with all other treatments (Fig. 6b), which was also consistent with the sharp decrease in its diversity. Taxonomic analysis revealed that ASV61 and ASV172 inoculation led to an increase in the relative abundance of *Gammaproteobacteria*, and a decrease in *Alphaproteobacteria* and *Acidobacteriota* (Fig. 6c). Focusing on the top 10 genera (Fig. 6d), we found that co-inoculation of either ASV61 or ASV172 with the pathogen significantly reduced pathogen abundance in the RS (*P* < 0.05). *Chryseobacterium* (ASV172) established a dominant presence, constituting over 55% of the community in the ASV172-alone treatment and 5.71% in the ASV172 + Rs treatment, significantly higher than in other treatments (relative abundance <0.01%), indicating its successful colonization and proliferation in the RS (Fig. 6d). In contrast, *Stenotrophomonas* (ASV61) exhibited limited colonization, remaining at low abundance across all treatments (relative abundance <0.2%). Interestingly, the Rs treatment showed a significant enrichment of *Stenotrophomonas* (Fig. 6e), suggesting a plant-mediated recruitment of this genus in response to pathogen infection. Moreover, inoculation with ASV61 or ASV172 promoted the enrichment of other potentially beneficial genera, such as *Paraburkholderia* and *Pseudomonas* (Fig. 6d), indicating these antagonistic strains may favor the proliferation of other beneficial members of the RS community.

Whole-genome sequencing of *Chryseobacterium* sp. ASV172 was performed to provide a preliminary genomic insight into its potential disease suppression mechanisms. The complete genome of *Chryseobacterium* sp. ASV172 consisted of a single chromosome, with a size of 4,664,037 bp and a GC content of 35.91% (Fig. S5a). Comparative genomic analysis showed the highest similarity of ASV172 to *C. wanjuense* (Fig. S5b). To identify potential antimicrobial compounds, putative secondary metabolites BGCs in *Chryseobacterium* sp. ASV172 were predicted using the antiSMASH software. The analysis revealed the presence of 12 putative BGCs, but only 3 (Clusters 1, 2, and 7) showed significant similarity to BGCs encoding known antibacterial substances (Table S7). Among the 3 BGCs, only Cluster 7 exhibited high similarity (80%) to BGCs responsible for flexirubin biosynthesis. A significant number (9 out of 12) of the predicted BGCs represented novel clusters without significant similarity to known secondary metabolite pathways. These novel BGCs may be responsible for synthesizing new compounds with potential antimicrobial activities.

### Antagonistic strains promoted plant fitness by enhancing plant defense system

**Figure 7:**
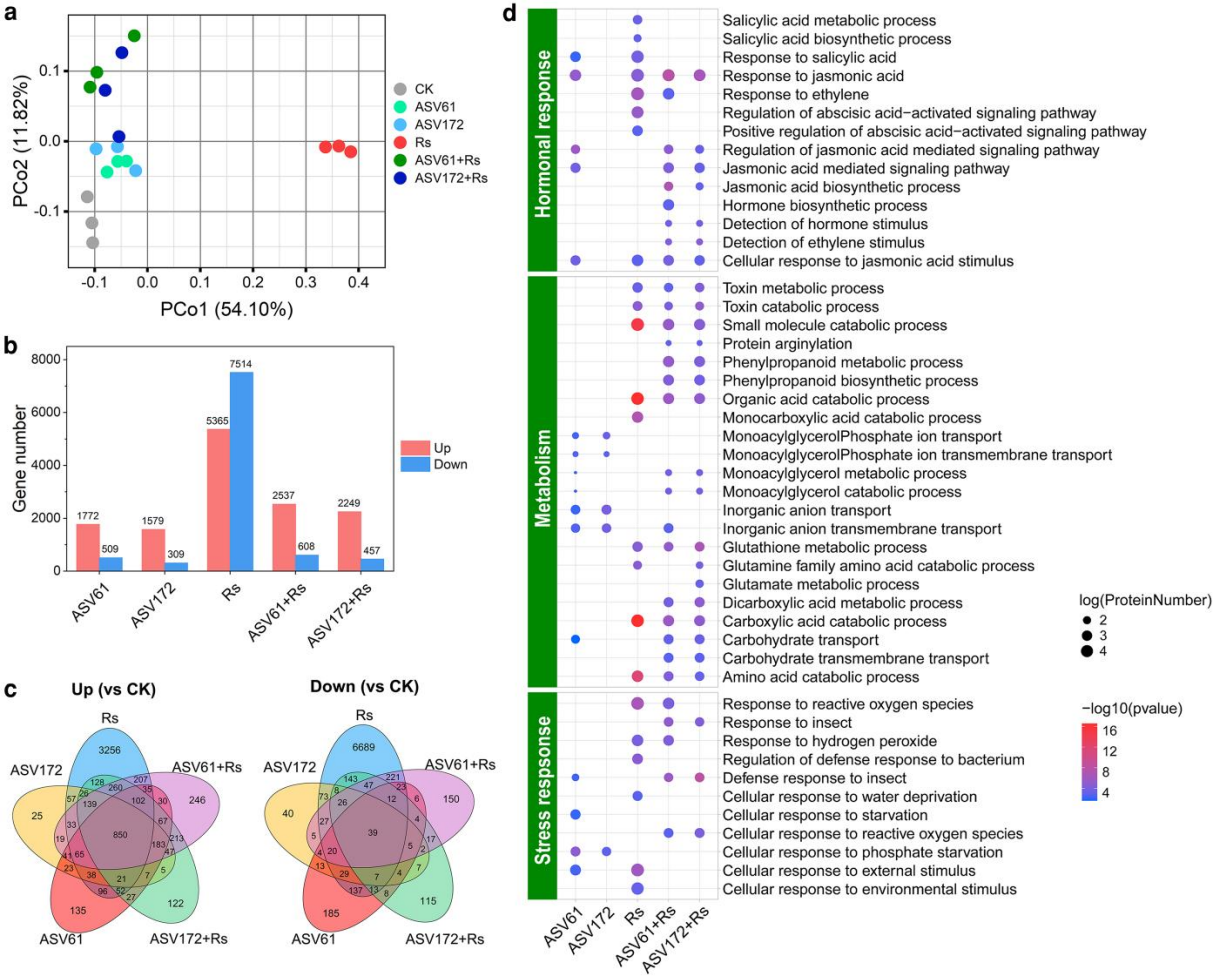
Effects of inoculation with antagonistic strains, *R. solanacearum* and co-inoculation (antagonistic strains and pathogen) on tobacco root transcriptome profiles compared with the control group at 10 d post *R. solanacearum* inoculation (10 dpi). a) PCoA based on Bray–Cutis dissimilarity matrices showing distinct transcriptome profiles among treatments. b) DEGs in tobacco roots across treatments compared with the control group. Bar plots represent the total number of up- and down-regulated genes. c) Venn diagrams depicting overlaps of up-regulated (left) and down-regulated (right) DEGs between treatments. d) GO enrichment analysis of significantly up-regulated DEGs (biological process), with bubble size representing gene count and color indicating enrichment significance (*P*-value).

To elucidate the molecular mechanisms underlying antagonistic bacteria-mediated enhancement of tobacco resistance, we performed transcriptome profiling of root tissues at 10 dpi. PCoA revealed 4 distinct clusters corresponding to the control (CK), *R. solanacearum* infection (Rs), antagonistic bacteria treatments (ASV61/ASV172), and co-inoculation treatments (ASV61 + Rs/ASV172 + Rs) (Fig. 7a). Compared with CK, *R. solanacearum* infection induced the largest number of DEGs (5,365 up, 7,514 down), followed by co-inoculation treatments (ASV61 + Rs: 2,537 up, 608 down; ASV172 + Rs: 2,249 up, 457 down) and antagonistic bacteria treatments (ASV61: 1,772 up, 509 down; ASV172: 1,579 up, 309 down) (Fig. 7b). Overlaps of DEGs between different treatments showed a substantial number of DEGs were unique to *R. solanacearum*-infected plants (Fig. 7c). All upregulated DEGs detected were used to explore the enriched biological processes in tobacco. GO enrichment analysis revealed significant enrichment of hormone-, metabolism-, and stress-related terms in Rs and co-inoculation plants (Fig. 7d and Table S8). Specifically, *R. solanacearum* activated SA- and abscisic acid (ABA)-related pathways, along with pathways related to environmental stresses (eg, hydrogen peroxide, bacterium, water deprivation and environmental stimulus). In contrast, co-inoculation with antagonistic bacteria strongly activated defense-related pathways associated with JA, ethylene, hormone signaling, and insect response. Furthermore, phenylpropanoid biosynthetic and metabolic process, monoacylglycerol catabolic and metabolic process, and carbohydrate transport pathways were specifically upregulated in co-inoculation plants, but not in *R. solanacearum*-infected plants (Fig. 7d).

**Figure 8:**
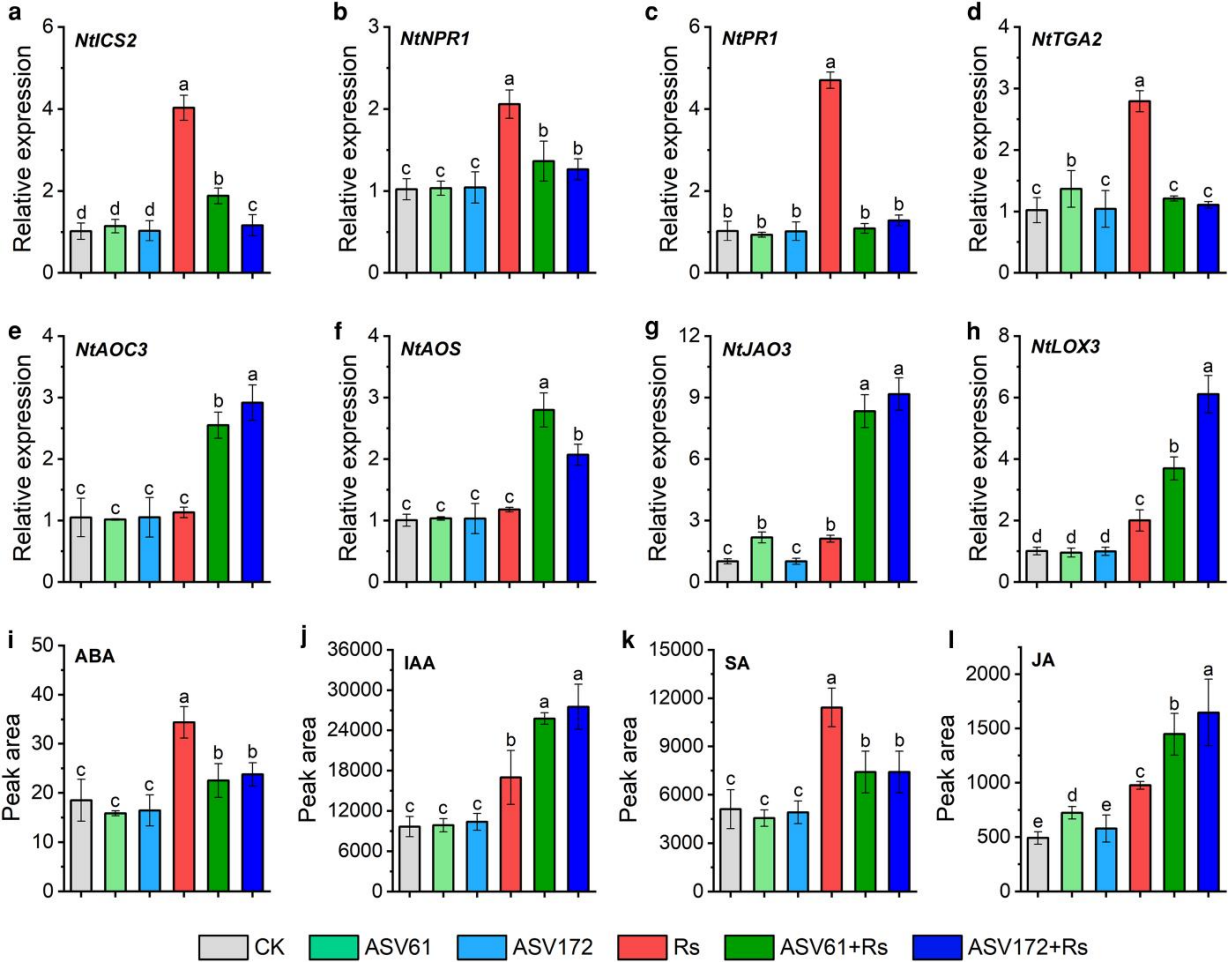
Effects of inoculation with antagonistic strains, *R. solanacearum* and co-inoculation (antagonistic strains and pathogen) on important genes expression and hormone content of tobacco roots. a to d) The expression of genes associated with SA signaling pathway at 10 d post-inoculation (10 dpi) with *R. solanacearum*. e to h) The expression of genes associated with JA signaling pathway at 10 dpi. i to l) The content of plant hormones, including ABA, IAA, SA and JA at 10 dpi. Different lowercase letters above bars indicated statistically significant differences (*P* < 0.05) as assessed by 1-way ANOVA followed by Tukey's multiple comparison test.

To validate the transcriptome findings and comprehensively delineate the temporal hormonal regulation, we quantified the expression of key genes associated with SA (*NtICS2*, *NtNPR1*, *NtPR1*, *NtTGA2*) and JA (*NtAOC3*, *NtAOS*, *NtJAO3*, *NtLOX3*) signaling pathways in tobacco roots at 2 and 10 dpi. At 2 dpi, *R. solanacearum* infection significantly induced the expression of both SA- and JA-pathway genes, with fold increases ranging from 2.12 to 14.79 and 2.14 to 7.55, respectively, compared with CK (Fig. S6). Notably, co-inoculation with antagonistic strains (ASV61 + Rs/ASV172 + Rs) further amplified the JA defense response, resulting in a 2.01- to 17.92-fold higher induction than *R. solanacearum*-alone infection (Fig. S6). By 10 dpi, a distinct shift was observed. In plants inoculated with *R. solanacearum* alone, SA-related genes remained elevated (2.02- to 4.58-fold higher than CK) (Fig. 8a to d). In contrast, significant induction of JA-responsive genes (2.06- to 9.10-fold higher than the control) was detected exclusively in the co-inoculation treatments (Fig. 8e to h), robustly confirming our RNA-seq data. Furthermore, phytohormone quantification at 10 dpi corroborated these transcriptional patterns. SA and ABA levels were highest in roots infected with *R. solanacearum* alone, followed by the co-inoculation treatments. Conversely, JA and indole-3-acetic acid (IAA) concentrations were significantly elevated in the co-inoculation treatments compared with all other treatments (Fig. 8i to l; *P* < 0.05).

## Discussion

Plants under the pathogen pressure employ various defense mechanisms, including actively reshaping their associated microbial communities. Accumulating evidence have indicated that plants can manipulate specific microbial communities to modify their phenotypes, enhance growth, and suppress disease (French et al. 2021; Santoyo 2022). This study systematically elucidated the niche-specific effects of BWD on plant microbiome assembly, revealing adaptive strategies of plants in recruiting beneficial microbes to counteract pathogen invasion. We discovered that BWD-induced microbiome restructuring exhibited spatial heterogeneity across different plant compartments, with the RP emerging as a critical interface mediating both pathogen resistance and the recruitment of beneficial microbes. The discovery of *Stenotrophomonas* sp. ASV61 and *Chryseobacterium* sp. ASV172 in the RP highlighted the potential of exploiting native plant-microbe interactions for targeted BWD management.

### Niche-specific impacts of BWD and microbial community differentiation

Microbial communities colonize virtually all plant tissues, including the soil–root continuum (RS/RP), internal plant tissues (root, stem, and leaf endosphere), and the air–plant interface (phyllosphere) (Trivedi et al. 2020). Each compartment establishes unique biotic and abiotic conditions that shape microbial composition and function. Our findings revealed the compartmentalized effects of BWD on microbial diversity and community structure (Fig. 1). The differential susceptibility among niches may be attributed to variations in nutrient availability, proximity to root exudates, or selective pressures exerted by the pathogen (Li et al. 2024). The observed gradient of decreasing alpha diversity from soil to inner plant tissues (Fig. S1a and b) aligned with established patterns of microbial filtering along the soil-plant continuum (Xiong et al. 2021; Cheng et al. 2024). While previous studies have often emphasized the pivotal role of the RS microbiome in disease suppression (Li et al. 2021), including evidence that early compositional divergence of RS microbiome could predict disease outcomes (Gu et al. 2022) and that RS microbiota from resistant tomato plants could transmit bacterial wilt suppression (Kwak et al. 2018), our results refined this spatial understanding by highlighting the RP as the compartment experiencing the most significant microbial community restructuring upon pathogen invasion (Figs. 1 and 2). This indicated that the RP, the immediate surface of the root, may be an even more critical battleground for pathogen-host-microbiome interactions than previously appreciated (Zhang et al. 2017). Moreover, this spatial refinement suggested that pathogen-induced microbiome remodeling may initiate at the root-soil contact zone before propagating to the RS community, underscoring the RP's unique position as an ecotone where host selection pressures and soil microbial dynamics converged most intensively.

Bacterial communities displayed greater sensitivity to BWD compared with fungal communities, particularly in the RP and RE (Figs. 1 and 2). The strong spatial consistency in bacterial response patterns across different sites (Fig. S1) implied that deterministic processes (eg, host-mediated selection) dominated bacterial community assembly under disease stress. Conversely, fungal communities exhibited greater stochasticity in their response to BWD, potentially reflecting their distinct life history strategies and ecological tolerances. The paradoxical increase of bacterial diversity in the diseased RP, contrasting with reduced diversity in other compartments (Fig. 1b), which suggested a potential high diversity of microbial composition in diseased RP, possibly driven by the plant's attempt to recruit beneficial microbes to combat the pathogen (Berg et al. 2017). Taxonomic profiling further verified BWD-induced compositional shifts, marked by increased abundances of *Alphaproteobacteria* and *Actinomycetota*, and *Tremellomycetes* in diseased RP (Fig. 2a and b). These taxa, often associated with stress tolerance or niche competition (Prathyusha and Bramhachari 2018; Vujanovic 2021; Hu et al. 2023), may exploit pathogen-mediated disruptions to colonize vacated ecological spaces.

### Host-mediated recruitment of beneficial microbes under pathogen stress

Plants under biotic stress often employ strategic recruitment of beneficial microbes to bolster defense (Liu et al. 2024). Our study provided multi-layered evidence supporting this ecological defense strategy. Although a substantial proportion of bacterial (68.51%) and fungal (70.31%) enriched ASVs were confined to single niches (Fig. 3c and f), the RP emerged as a key reservoir for functionally relevant antagonists. The limited overlap of bacterial (26.41%) and fungal (35.49%) ASVs between healthy and diseased RP communities (Fig. 3a and d), coupled with the highest number of niche-specific enriched ASVs in the diseased RP (Fig. 3c and f), suggested a targeted, host-mediated selection process rather than stochastic community shifts (Luo et al. 2025). This compartmentalized enrichment pattern likely reflected localized root exudate reprogramming (Yang et al. 2025), similar to the metabolite-guided functional activation of *Bacillus velezensis* SQR9 during cucumber development (Feng et al. 2023), where specific signaling molecules (eg, phenolic acids) may selectively enrich antagonists while suppressing pathogens.

To functionally validate these observations, we isolated cultivable bacteria from the diseased RP (Fig. 4a) and performed plate confrontation assays (Fig. 4b). These assays provided direct evidence for plant-mediated recruitment of beneficial microbes. The 8 antagonistic strains identified were significantly enriched in the diseased RP, and, importantly, most showed a positive correlation with *Ralstonia* abundance (Fig. 4d). This correlation, also observed with endophytic microbes (Tao et al. 2024), indicated a potential plant-mediated recruitment mechanism triggered by the pathogen. Specifically, the strong antagonistic activities of *Stenotrophomonas* sp. ASV61 and *Chryseobacterium* sp. ASV172, along with their significant positive correlations with *Ralstonia*, highlighted their potential as key players in plant defense against bacterial wilt. Crucially, the convergence of amplicon sequencing data with culturable antagonistic isolates provided compelling evidence that structural community shifts translated to functional disease suppression—a critical advancement in understanding plant-microbe-pathogen tripartite interactions (Li et al. 2022).

However, it is noteworthy that the pronounced plant growth-promoting (PGP) traits observed for ASV61 and ASV172 in vitro (Fig. S3) did not translate into significant growth promotion in planta under our experimental conditions, aside from a modest effect on plant height (Fig. 5). This divergence was consistent with existing reports indicating that the performance of rhizobacterial under axenic conditions often poorly predicted field efficacy due to complex biotic or abiotic interactions. Several factors likely contributed to this observed divergence. The complex soil-plant-microbe environment in pots may limit the expression or efficacy of bacterial PGP traits (Orozco-Mosqueda et al. 2022). Furthermore, the disease pressure and nutrient conditions in our study may have prioritized pathogen suppression over growth enhancement. Despite the minimal direct PGP effects observed in planta, the robust disease suppression conferred by ASV61 and ASV172, particularly ASV172, clearly demonstrated their value as protective agents for tobacco. Future studies could investigate the colonization dynamics and molecular mechanisms underlying their dual roles in disease control and potential growth promotion under stress conditions.

### Biocontrol mechanisms of antagonistic strains

Antagonistic strains of *Stenotrophomonas* sp. ASV61 and *Chryseobacterium* sp. ASV172 represented 2 phylogenetically distinct taxa with contrasting ecological strategies in plant protection. Crucially, both strains were isolated from the RP microbiome specifically enriched under BWD stress, indicating that disease pressure actively shapes a microbial community poised for defense (Noman et al. 2021). Their increased presence and abundance on the RP during infection are therefore not incidental but likely a hallmark of the plant's recruitment strategy to counteract the pathogen (Yang et al. 2025). *Stenotrophomonas*, belonging to the *Gammaproteobacteria*, is a metabolically versatile genus known for its ability to degrade a wide range of organic compounds and its presence in diverse environments, including soil, water, and plant tissues (Ryan et al. 2009; Jia et al. 2019; Niu et al. 2023). *Stenotrophomonas* species, particularly *S. maltophilia*, have been extensively studied for their biocontrol activity against various plant pathogens (Fariman et al. 2022; Sharma et al. 2024). Similarly, *Chryseobacterium*, belonging to the family *Flavobacteriaceae*, are also abundant in diverse environments, including soil, water, plants, and animals (Montero-Calasanz et al. 2013; Kämpfer et al. 2014; Zhang et al. 2021b). Although some *Chryseobacterium* species have also been reported as biocontrol agents (Domenech et al. 2006; Suryadi et al. 2019), the research on their biocontrol potential is generally less extensive compared with *Stenotrophomonas*. Our findings highlighted the potential of *Chryseobacterium* sp. ASV172 as a particularly promising biocontrol agent, exhibiting superior disease suppression compared with *Stenotrophomonas* sp. ASV61 (Fig. 5). This difference in efficacy, despite both strains demonstrating significant biocontrol activity, underscored the importance of exploring the diversity of biocontrol agents within less-studied genera like *Chryseobacterium*.

The biocontrol and PGP mechanisms employed by ASV61 and ASV172 appeared to be multifaceted, involving both direct antagonism and indirect promotion of plant health. The superior colonization ability of *Chryseobacterium* sp. ASV172 (Fig. 6) likely underpinned its enhanced biocontrol efficacy, enabling it to occupy a critical niche, outcompete the pathogen for resources, and establish a protective microbial shield on the root surface. This competitive exclusion, combined with its potential production of antibacterial flexirubin (Azman et al. 2018), suggested a direct inhibitory effect on *R. solanacearum*. In contrast, *Stenotrophomonas* sp. ASV61 may rely more heavily on indirect mechanisms, such as modulating the RS microbiome to favor beneficial microbes (Fig. 6) and/or ISR in the host plant (Fig. 7). Furthermore, the PGP activity of both strains, evidenced by plant growth promotion, is intrinsically linked to their ability to modulate plant hormone pathways, as indicated by RNA-seq.

Transcriptomic and temporal gene expression analyses revealed that both biocontrol strains augmented plant defense by dynamic regulation of phytohormone signaling. During early infection (2 dpi), *R. solanacearum* triggered a broad upregulation of both SA and JA signaling markers, with JA -responsive genes further amplified in co-inoculated plants (Fig. S6). This early broad-spectrum activation, potentially reflecting a general pathogen-triggered immune response (Hönig et al. 2023), contrasted with previous findings that *Ralstonia* suppressed SA production and signaling while disrupting JA pathways (Bentham et al. 2020; Chachar et al. 2025). We proposed this discrepancy likely stemmed from differences in the timing of analysis and the specific pathological context. Nevertheless, by the later stage (10 dpi), the pathogen-alone treatment sustained SA related responses, while JA-responsive genes were significantly induced only in the presence of antagonistic strains (Figs. 7 and 8). This suggested that *R. solanacearum* might manipulate the host by favoring SA signaling, while the biocontrol strains countered this by reactivating JA-mediated defense, thereby creating a defense profile less favorable to the pathogen. Such hormonal rebalancing likely contributed to the observed biocontrol efficacy, even amid ongoing disease progression. This shift suggested that the PGP rhizobacteria (PGPR) strains actively redirected the host's defense posture from a pathogen-manipulated, SA-persistent state toward a robust JA-mediated defense, which was often more effective against necrotrophic phases of the *Ralstonia* infection cycle and linked to the restoration of growth-defense balance (Marqués-Gálvez et al. 2025). This hormonal rebalancing, a key aspect of defense priming by beneficial microbes, likely contributed significantly to their observed biocontrol and growth-promotion efficacy (Zhang et al. 2026). Beyond hormonal shifts, co-inoculation also enhanced processes related to cell wall biogenesis and oxidative stress mitigation, thereby strengthening physical and biochemical barriers against pathogen invasion (Underwood 2012; Jian et al. 2024). Therefore, RNA-seq results directly connected the PGPR activity of ASV61 and ASV172 to their profound influence on host hormone-regulatory pathways, explaining how they concurrently promoted growth and prime for a more effective defense.

However, the specific elicitors produced by each strain, the precise signaling pathways activated, and the downstream defense responses induced may differ, which may contribute to the observed differences in biocontrol efficacy. Future research aimed at identifying strain-specific antimicrobial compounds, characterizing RS interactions, and detailing plant spatiotemporal defense responses will provide a more comprehensive understanding for optimizing biocontrol strategies.

**Figure 9:**
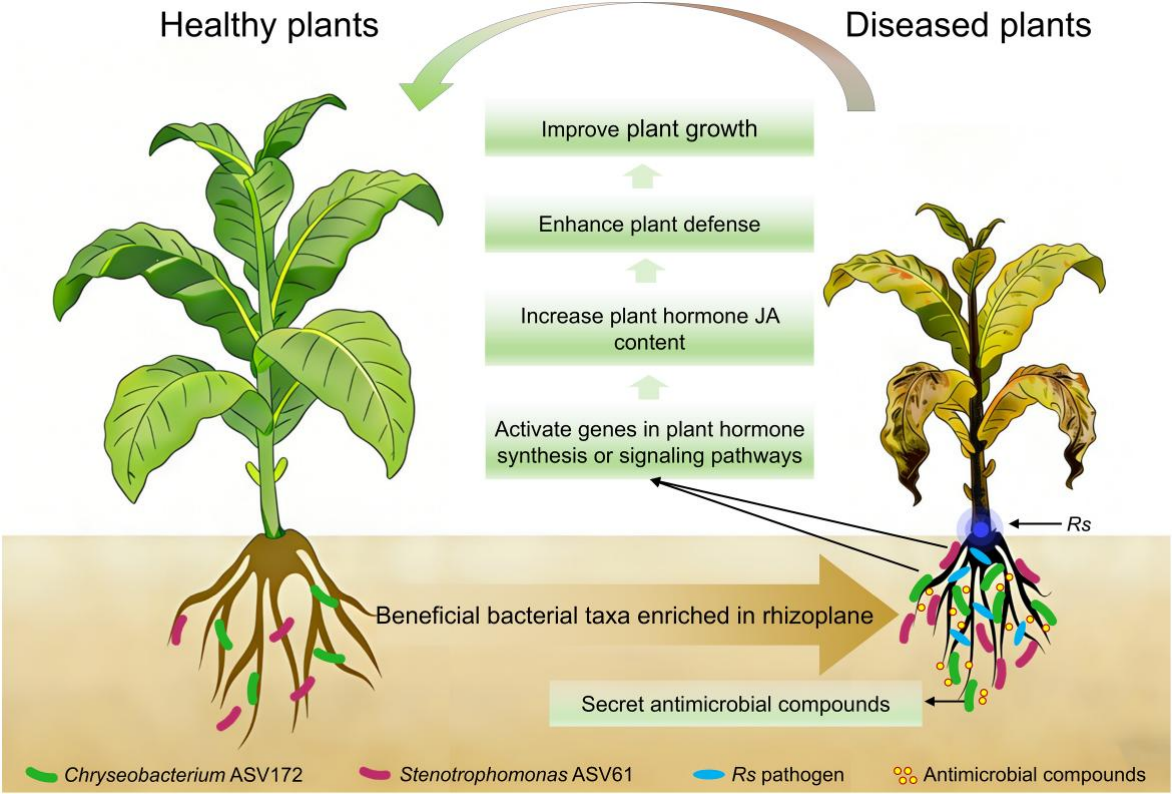
Schematic diagram demonstrating the potential benefits of RP bacteria *Chryseobacterium* sp. ASV172 and *Stenotrophomonas* sp. ASV61 on plant growth and defense. When exposed to *R. solanacearum*, the tobacco plant's RP experiences an enrichment of beneficial bacteria, including ASV172 and ASV61. These beneficial bacteria can promote plant growth and increase disease resistance by activating robust JA signaling pathway and secreting antimicrobial compounds.

In summary, our study demonstrated niche-specific microbiome alterations induced by BWD, identifying the RP as a key site for beneficial microbial recruitment and defense coordination. When exposed to *R. solanacearum*, the tobacco plant's RP experienced an enrichment of beneficial bacteria, including ASV172 and ASV61. We provided evidence that these beneficial strains isolated from the tobacco RP contributed to improving plant growth and enhancing disease suppression, primarily by modulating the host's hormone signaling pathways. While *Ralstonia* alone manipulated host defenses by sustaining SA responses, antagonistic strains re-directed the plant toward robust JA signaling, thereby restoring a more effective defense posture (Fig. 9). These findings underscored the RP's critical importance for building resilient microbiomes against soil-borne diseases, enabling targeted RP microbiome engineering for sustainable BWD management.

## Supplementary Material

kiag483_Supplementary_Data

## Data Availability

The raw sequence data are available in the Genome Sequence Archive in National Genomics Data Center, Beijing Institute of Genomics, Chinese Academy of Sciences (https://bigd.big.ac.cn/gsa), under accession number CRA026610 and CRA026611 (16S and ITS data of different niches), CRA027934 (Rhizosphere microbiome data with antagonistic strains inoculation), CRA027794 (Transcriptome data with antagonistic strains inoculation).
