# Supplementary material for: Rhizoplane microbiome: niche-specific recruitment and plant defense priming against bacterial wilt disease
Source: Plant Physiol. 2026 Jul 8;201(3):kiag483. doi: 10.1093/plphys/kiag483 (PMC13418362; doi:10.1093/plphys/kiag483)
Supplement: kiag483_Supplementary_Data [file kiag483_supplementary_data.zip › Supplementary Figures.docx]

**Supplementary Figures**


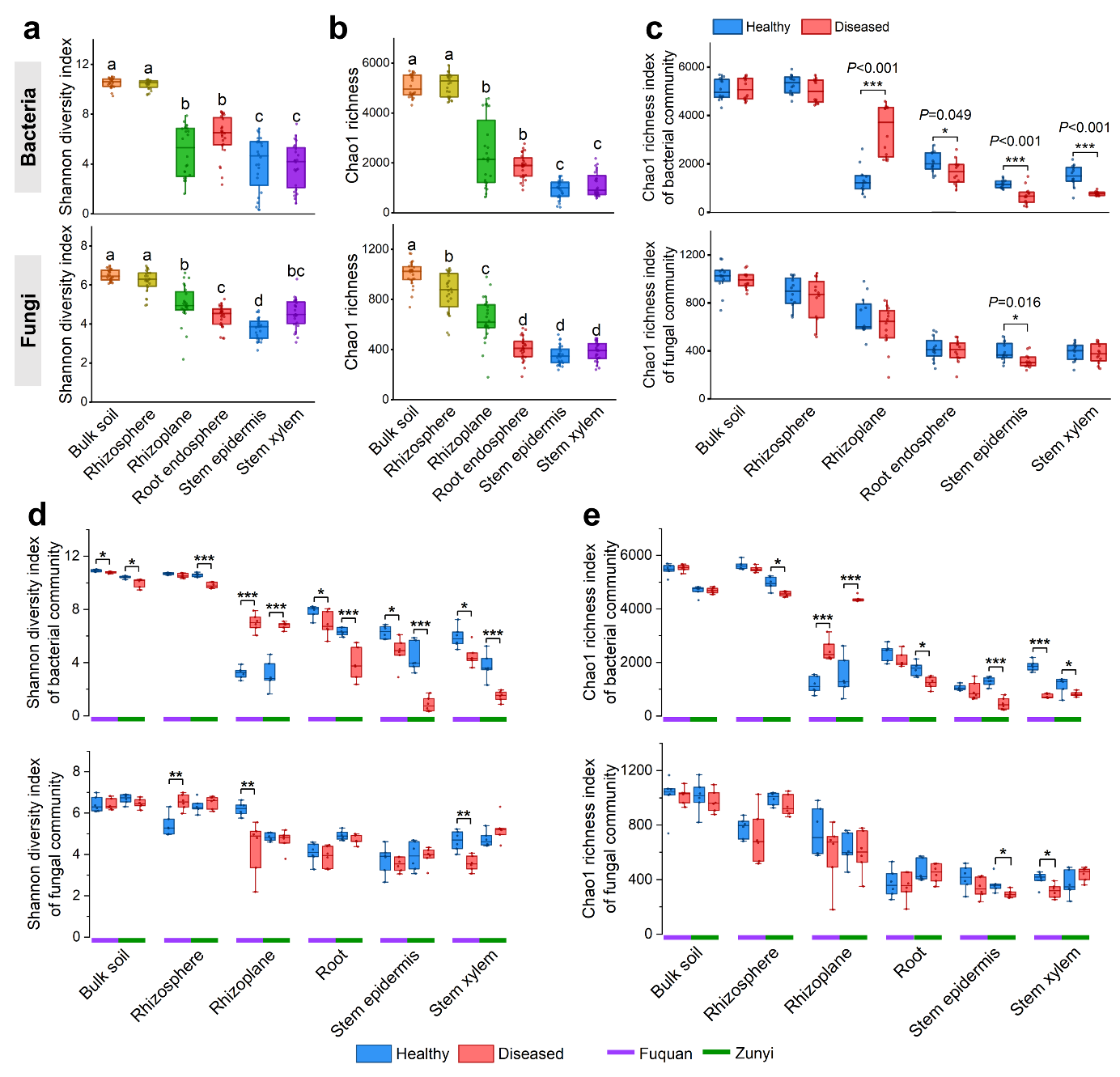


**Fig. S1** Changes in alpha diversity indices of bacterial and fungal communities. Changes in Shannon diversity **(a)** and Chao1 richness indices **(b)** of bacterial and fungal communities in different compartments. **(c)** Chao1 richness index of bacterial and fungal communities in six compartments of healthy (blue) and diseased (red) plants. Changes in Shannon diversity **(d)** and Chao1 richness indices **(e)** in six compartments of healthy and diseased plants at two study sites (Fuquan and Zunyi).


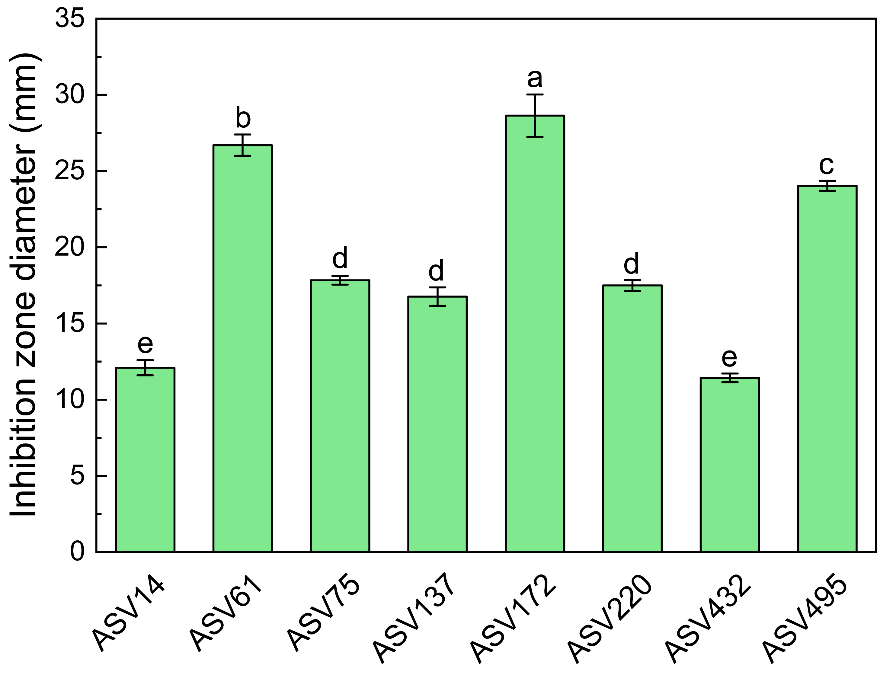


**Fig. S2** The inhibition zone diameters of eight isolated bacterial strains against pathogen *R. solanacearum*. Different letters above the columns indicate significant differences at *P* < 0.05 by ANOVA with an LSD test.


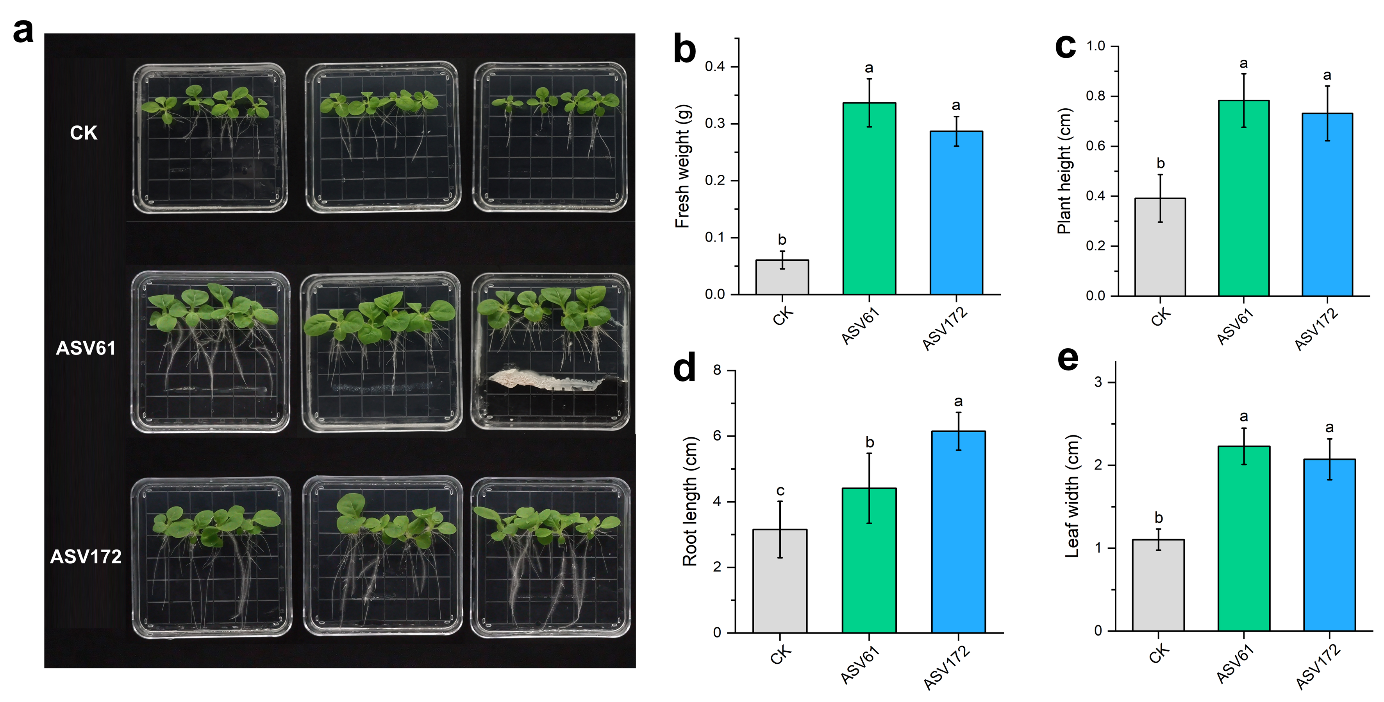


**Fig. S3** The growth promotion effect of antagonistic strains *Stenotrophomonas* sp. ASV61 and *Chryseobacterium* sp. ASV172. **(a)** Phenotype of tobacco seedlings after inoculation with ASV61 and ASV172 on MS plates. Distilled water was used as control. Column charts showing growth parameters of tobacco seedlings, including aboveground fresh weight **(b)**, plant height **(c)**, root length **(d)** and leaf width **(e)**. Different letters above the columns indicate significant differences at *P* < 0.05 by ANOVA with an LSD test.


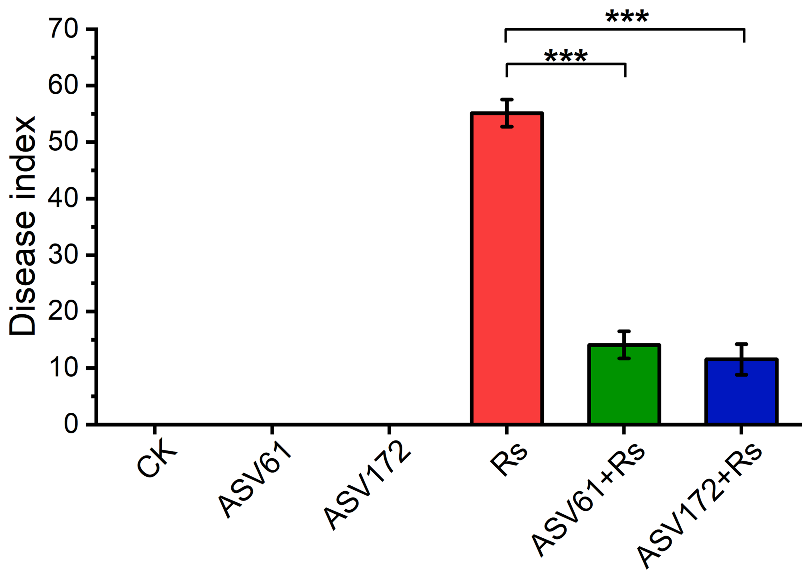


**Fig. S4** Disease index of tobacco plants under different treatments. CK: no inoculation; ASV61: inoculation with ASV61 only; ASV172: inoculation with ASV172 only; Rs: inoculation with *R. solanacearum* only; ASV61+Rs: inoculation with ASV61 and *R. solanacearum*; ASV172+Rs: inoculation with ASV172 and *R. solanacearum.* Asterisks indicate significant differences between treatments (***, *P* < 0.001).


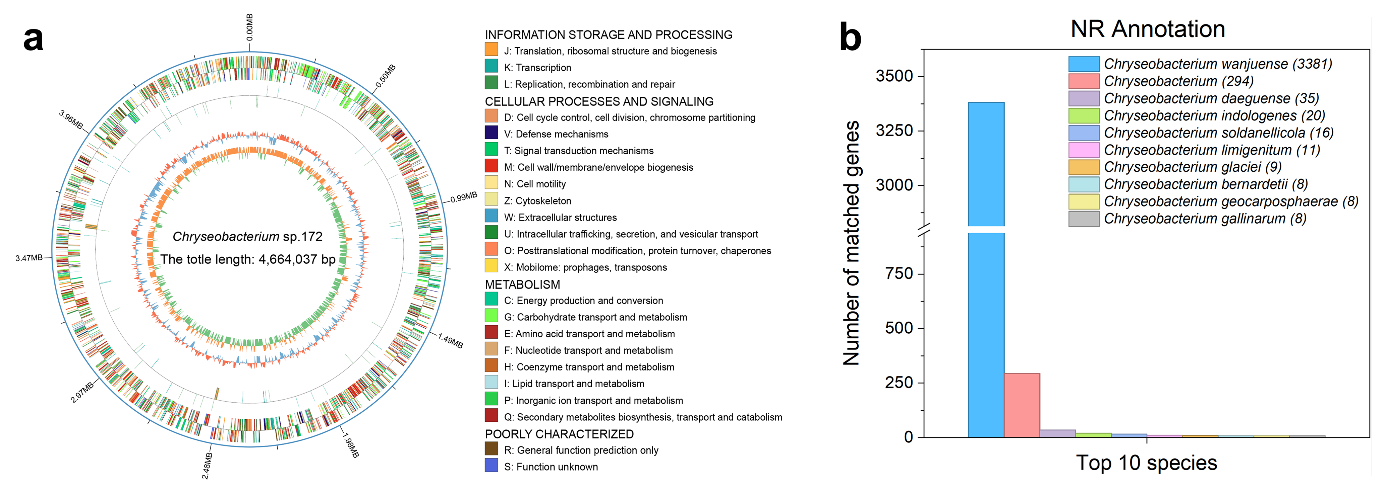


**Fig. S5** Genomic overview and taxonomic annotation of *Chryseobacterium* sp. ASV172. **(a)** Circular genome map of ASV172. **(b)** Summary of taxonomic and functional annotations of ASV172. Bar plots showing the number of annotated species and gene.


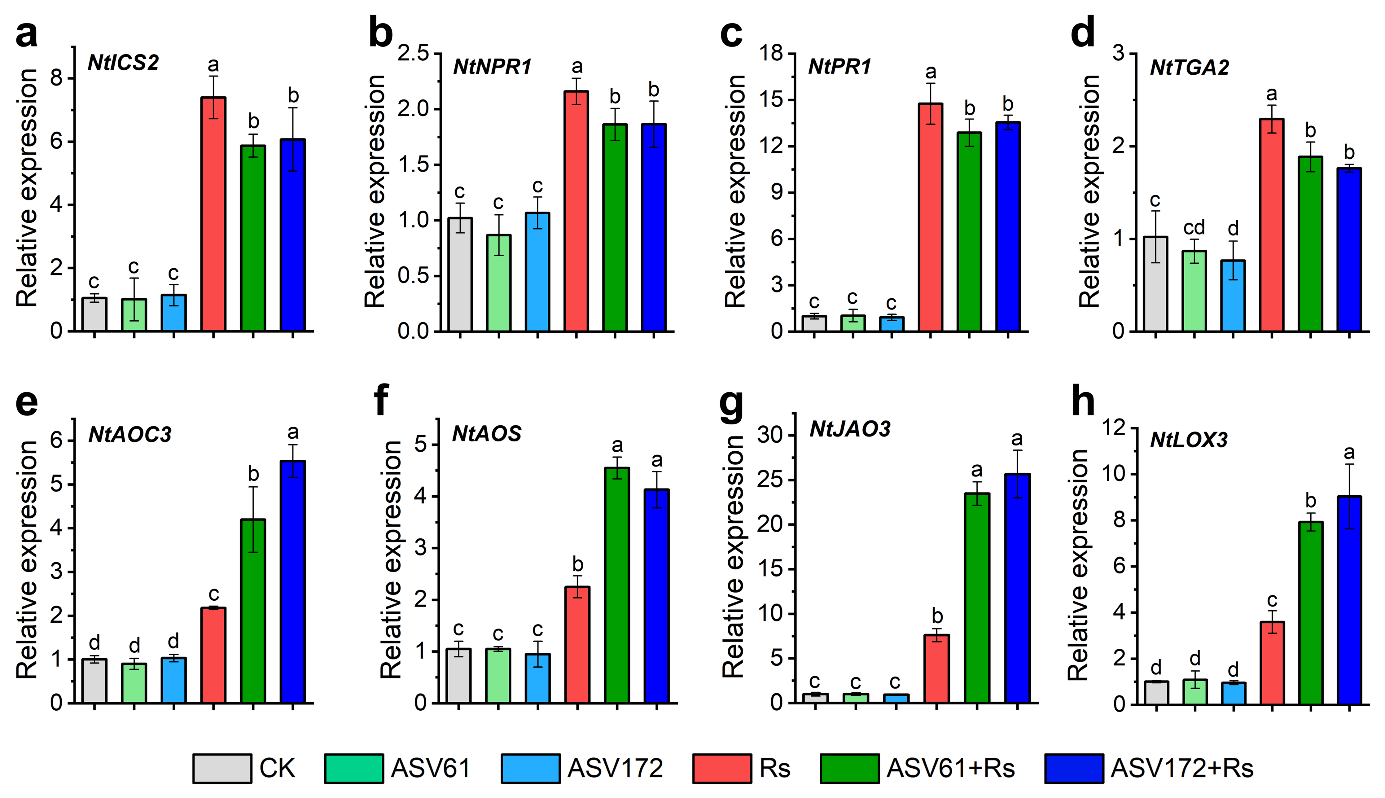


**Fig. S6** Effects of inoculation with antagonistic strains, *R. solanacearum* and co-inoculation (antagonistic strains and pathogen) on the expression of important genes associated with salicylic acid (SA) **(a-d)** and jasmonic acid (JA) **(e-h)** signaling pathways at 2 days post*-*inoculation (2 dpi) with *R. solanacearum*.
